# New Steroidal Saponins from the Rhizomes of *Paris vietnamensis* and Their Cytotoxicity

**DOI:** 10.3390/molecules23030588

**Published:** 2018-03-06

**Authors:** Yang Liu, Minchang Wang, Ke Liu, Pengcheng Qiu, Shan Zhang, Yunyang Lu, Na Tang, Haifeng Tang

**Affiliations:** 1 Institute of Materia Medica, School of Pharmacy, Fourth Military Medical University, Xi’an 710032, China; so870823@163.com (Y.L.); qpc023@126.com (P.Q.); zhangshan9815@163.com (S.Z.); luyunyanggq@163.com (Y.L.); donnao7@163.com (N.T.); 2 State Key Laboratory of Fluorine & Nitrogen Chemicals, Xi’an 710065, China; wmc204@163.com (M.W.); happycoco5133@163.com (K.L.); 3 Xi’an Modern Chemistry Research Institute, Xi’an 710065, China

**Keywords:** *Paris vietnamensis*, steroidal saponins, spirostanol saponins, cytotoxicity

## Abstract

Four new spirostanol saponins, named pavitnosides A–D (**1**–**4**), with six known steroidal saponins **5**–**10** were isolated from the rhizomes of *Paris vietnamensis*. Their chemical structures were determined based on extensive spectroscopic studies and chemical methods. The aglycones of pavitnoside B and pavitnoside C were not reported in previous work. The cytotoxicity of all saponins was evaluated against human glioblastoma U87MG and U251 cell lines. The new spirostanol saponin **1** displayed weak anti-proliferative activity against U87MG cell line and the known saponins **8** and **9** exhibited significant cytotoxicity against the two tumor cell lines, with IC_50_ values of 2.16 to 3.14 μM, but did not affect the growth of primary cultures of human astrocytes.

## 1. Introduction

**Figure 1:**
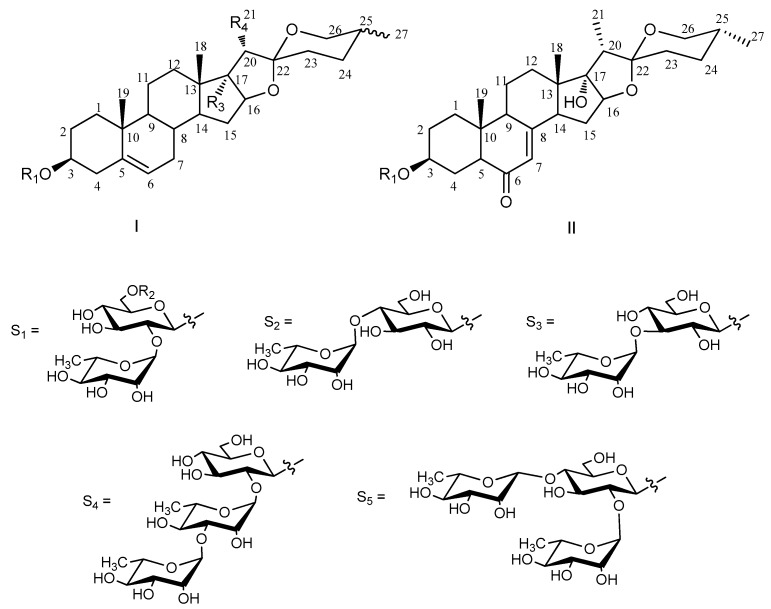
Structures of Compounds **1–10** from *Paris vietnamensis*.

The genus *Paris* (Liliaceae) has been used as a traditional Chinese medicine for a long time. In the Chinese Pharmacopoeia, *Rhizoma Paridis*, recorded as the rhizomes of *Paris polyphylla* var. *yannanensis* and *Paris polyphylla* var. *Chinensis* [1], are widely used as folk medicine for heat-clearing and detoxifying, relieving swelling and pain, sore throat, snake bites and traumatic injure [2]. By extensive phytochemical and pharmacological studies, steroidal saponins have proven to be the chief active ingredients. Their significant bioactivities, including antitumor, anti-inflammatory, anti-melanogenic, antifungal, antiulcerogenic and hemostatic, have been confirmed in recent years [3,4,5,6,7,8,9,10,11]. As perennial plants, the domestic production of *Rhizoma Paridis* is insufficient, thus demand exceeds supply [2]. *P. vietnamensis* was chosen to investigate its chemical characteristics, aimed to replace *Rhizoma Paridis* and alleviate resource pressure. *P. vietnamensis* is mainly distributed in Guangxi, Yunnan province, China and North Vietnam. Its ideal growing environment is evergreen broad-leaved forest under 2000 m above sea level [12]. Up to now, one new and ten known steroidal saponins have been identified from the rhizomes of *P. vietnamensis*, and several compounds exhibit cytotoxic bioactivities [13]. These results prompted to continue further investigation on *P. vietnamensis* to discover novel structures and bioactive saponins. This paper describes the isolation and structural identification of four new spirostanol saponins and six known saponins (Figure 1), as well as the exploration of their in vitro cytotoxic activities.

## 2. Results and Discussion

**Figure 2:**
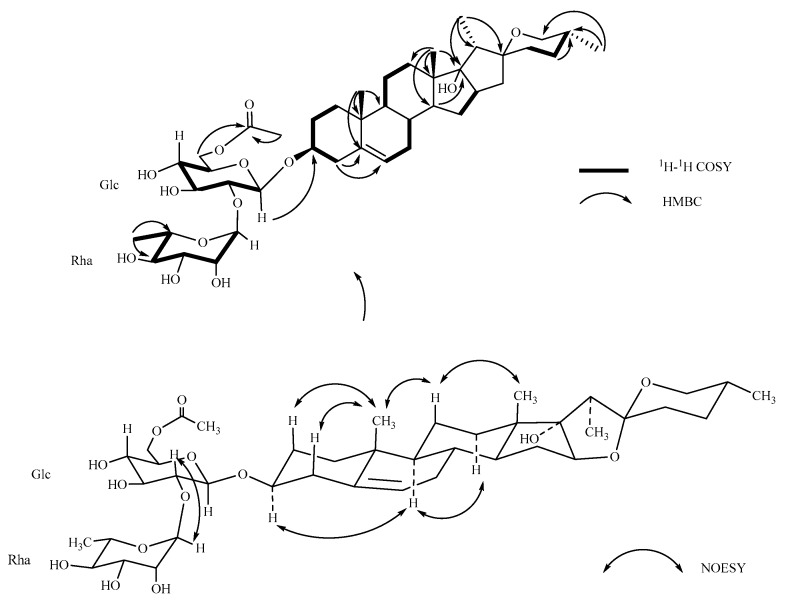
Key ^1^H-^1^H COSY, HMBC and NOESY correlations of Compound **1**.

Pavitnoside A (**1**), yellowish amorphous solid, was positive to Liebermann–Burchard and Molisch chemical reactions. The HR-ESI-MS exhibited a pseudomolecular ion peak at *m*/*z* 803.4170 [M + Na]^+^ (calcd. for C_41_H_64_O_14_Na, 803.4194), corresponding to the molecular formula of C_41_H_64_O_14_, corroborated by analysis of ^13^C-NMR and DEPT spectral data. The ^1^H-NMR spectrum of **1** showed four methyl groups at *δ_H_* 0.84 (3H, s, H-18), 1.05 (3H, s, H-19), 0.80 (3H, d, *J* = 6.4 Hz, H-27) and 0.90 (3H, d, *J* = 7.2 Hz, H-21) as well as one olefinic methine proton at *δ_H_* 5.40 (1H, br s, H-6), which suggested a steroidal skeleton [14,15]. Additionally, an oxymethylene proton (2H, m, H-26) was observed at *δ_H_* 3.34 and 3.49. The ^13^C-NMR spectrum of **1** revealed four angular methyl signals at *δ_C_* 17.67 (C-18), 19.98 (C-19), 9.26 (C-21) and 17.64 (C-27), one trisubstituted double bonds at *δ_C_* 142.03 (C-5) and 122.80 (C-6). Moreover, one quaternary carbon at *δ_C_* 110.10 (C-22) is a characteristic hemiacetal signal of the spirostanol aglycone [14]. In the HMBC spectrum, the cross peaks between H-4 (*δ_H_* 2.30 and 2.45) and C-5 (*δ_C_* 142.03)/C-6 (*δ_C_* 122.80), between H-19 (*δ_H_* 1.05) and C-5 (*δ_C_* 142.03) deduced that the double bond was located at C-5/C-6 (Figure 2). The α configuration of C-17 was deduced by the chemical shifts of *δ_C_* 90.68 (C-16) and 91.45 (C-17) [16,17,18]. The *β* configuration of C-3 was deduced by NOESY spectrum that correlation between H-3 (*δ_H_* 3.53) and H-9 (*δ_H_* 0.95) indicated H-3 in *α*-orientation. The 25*R* configuration was confirmed by the difference chemical shifts between H-26a and H-26b (Δab = 0.15 < 0.48) [19,20,21]. Considering these data and the reported literature [22], the aglycone of Compound **1** was identified as 25(*R*)-spirost-5-en-3*β*,17*α*-diol.

The ^13^C-NMR spectrum exhibited 41 signals, of which 27 were assigned to the aglycone moiety and 14 to the saccaride moiety. Acid hydrolysis of Compound **1** yielded d-glucose (Glc) and l-rhamnose (Rha) in a ratio of 1:1 by comparing the retention times with the corresponding authentic samples. The ^1^H-NMR spectrum of **1** showed two anomeric proton signals at *δ_H_* 4.51 (d, *J* = 7.76 Hz, H-1 of Glc) and *δ_H_* 5.20 (br s, H-1 of Rha). Correspondingly, the correlations in HSQC of anomeric carbon signals were revealed at *δ_C_* 101.04 and *δ_C_* 102.33, respectively. The sequence of all proton signals in monosaccharide was identified by combined use of ^1^H-^1^H COSY and TOCSY spectra. Then, the relative carbon signals were delineated through HSQC experiments (Table 1). In additional, one keto-methyl and one carbonyl carbon signals were observed at *δ_C_* 20.89 (3H, s, *δ_H_* 2.05) and 172.89. The up-field shifts of the keto-methyl (*δ_H_* 2.05) and Glc H-6 (*δ_H_* 4.20 and 4.35) implied that they may link the carbonyl group (*δ_C_* 172.89). In HMBC spectrum, the hypothesis was authenticated with long-rang correlations from H of keto-methyl (*δ_H_* 2.05)/Glc H-6 (*δ_H_* 4.20 and 4.35) to C-carbonyl (*δ_C_* 172.89). Moreover, the sequence of a disaccharide chain was confirmed by the cross peaks between Rha H-1 (*δ_H_* 5.20) and Glc C-2 (*δ_C_* 79.07) and the correlation from Glc H-1 (*δ_H_* 4.51) to C-3 (*δ_C_* 80.09) demonstrated the location of sugar linkage. This conclusion was supported by the NOESY spectrum exhibited in Figure 2. The *β* configurations of d-glucopyranosyl was deduced by anomeric proton coupling constants (*J* = 7.6 Hz > 7.0 Hz), the *α* anomeric configuration of l-rhamnopyranosyl was confirmed by the chemical shifts of Rha C-5 at *δ_C_* 69.91 [23,24]. Thus, the structure of Compound **1** was elucidated as 25(*R*)-spirost-5-en-3*β*,17*α*-diol-3-*O*-*α*-l-rhamnopyranosyl-(1→2)-6-acetyl-*β*-d-glucopyranoside, named Pavitnoside A (**1**).

Pavitnoside B (**2**), white amorphous solid, was positive to Liebermann–Burchard and Molisch chemical reactions. The HR-ESI-MS showed a pseudomolecular ion peak at *m*/*z* 777.4044 [M + Na]^+^ (calcd. for C_39_H_62_O_14_Na, 777.4037), corresponding to the molecular formula of C_39_H_62_O_14_, corroborated by analysis of ^13^C-NMR and DEPT spectral data. In ^1^H-NMR spectrum of **2**, three methyl signals were observed at *δ_H_* 0.86 (3H, s, H-18), 1.06 (3H, s, H-19) and 0.79 (3H, d, *J* = 6.4 Hz, H-27) as well as one olefinic methine proton at *δ_H_* 5.39 (1H, br s, H-6). The ^13^C-NMR spectrum of **2** exhibited corresponding carbon signals at *δ_C_* 17.34 (C-18), 19.99 (C-19) and 17.62 (C-27), one trisubstituted double bonds at *δ_C_* 142.06 (C-5) and 122.75 (C-6). Compared to **1**, one angular methyl at *δ_C_* 9.26 (C-21) was absent and the chemical shift of C-20 (*δ_C_* 53.15) was up-field (Δ*δ_C_* + 7.47 ppm), which indicated that a hydroxyl group substituted at the C-21 primary carbon atom. It was evidenced by ^1^H-^1^H COSY correlations between H-20 (*δ_H_* 2.25, dd, *J* = 5.84, 8.56 Hz) and H-21 [*δ_H_* 3.61 (m), 3.80 (m)] and by HMBC cross-peaks between H-21 (*δ_H_* 3.61, 3.80) and C-20 (*δ_C_* 53.15)/C-22 (*δ_C_* 110.17), between H-20 (*δ_H_* 2.25) and C-13 (*δ_C_* 46.25)/C-21 (*δ_C_* 59.55)/C-17 (*δ_C_* 91.45)/C-22 (*δ_C_* 110.17) (Figure S2). The double bond located at C-5/C-6 also confirmed by HMBC correlations between H-19 (*δ_H_* 1.06) and C-5 (*δ_C_* 142.06), between H-6 (*δ_H_* 5.39) and C-4 (*δ_C_* 39.66)/C-7 (*δ_C_* 33.83)/C-10 (*δ_C_* 38.15) (Figure S2). By comparison of **1**, the configuration of C-17 was *α*-orientation and the *β*-configuration of C-3 was also evidenced by NOESY spectrum. The chemical shifts between H-26a and H-26b (Δab = 0.13 < 0.48) decided the 25*R* configuration [25]. Based on above evidence, the aglycone of Compound **2** was established as 25(*R*)-spirost-5-en-3*β*,17*α*,21-triol. The C_27_ steroids aglycone of Compound **2** has not been reported in previous work. 

GC analysis after acid hydrolysis of Compound **2** demonstrated the same monosaccharides as **1**, i.e., presence of d-glucose and l-rhamnose. Compared to Compound **1**, Compound **2** also obtained the same linkages of sugar moieties. The only difference was that the signals of 6-acetyl connected with *β*-d-glucose were not observed in ^1^H-NMR and ^13^C-NMR spectrum. Thus, Compound **2** was characterized as 25(*R*)-spirost-5-en-3*β*,17*α*,21-triol-3-O-*α*-l-rhamnopyranosyl-(1→2)-*β*-d-glucopy ranoside, named Pavitnoside B (**2**).

Pavitnoside C (**3**), white amorphous solid, was positive to Liebermann–Burchard and Molisch chemical reactions. The molecular formula was established as C_39_H_60_O_14_ from the [M + Na]^+^ ion at *m*/*z* 775.3884 [M + Na]^+^ (calcd. for C_39_H_60_O_14_Na, 775.3881) in the HR-ESI-MS and the ^13^C NMR spectral data. The ^1^H-NMR spectrum of **3** exhibited one olefinic methine proton at *δ_H_* 5.73 (1H, br s, H-7). Four angular methyl signals at *δ_H_* 0.84 (3H, s, H-18), 1.25 (3H, s, H-19), 0.90 (3H, d, *J* = 7.2 Hz, H-21) and 0.86 (3H, d, *J* = 6.3 Hz, H-27) implied that **3** also possessed a steroidal skeleton [10,11]. Correspondingly, one trisubstituted olefinic group at *δ_C_* 126.59 (C-7) and 169.05 (C-8) and four angular methyl signals at *δ_C_* 17.81 (C-18), 17.83 (C-19), 9.45 (C-21), 17.64 (C-27) were discovered in the ^13^C-NMR. A characteristic quaternary carbon at *δ_C_* 111.11 (C-22) was assigned in Compound **3** to a spirostanol aglycone. Compared to **1**, C-6 was displaced by a ketone carbon at *δ_C_* 204.57, and the double bond changed to C-7/C-8. In HMBC spectrum, the cross peaks from *δ_Ha_* 2.48, *δ_Hb_* 2.73 (H-4)/*δ_H_* 2.49 (H-5) to *δ_C_* 204.57 (C-6) confirmed the ketone location. The double bond Δ^7(8)^ was deduced by correlations between *δ_H_* 5.73 (H-7) and *δ_C_* 46.62 (C-5)/*δ_C_* 40.08 (C-10), between *δ_H_* 2.03(H-14)/*δ_H_* 1.25 (H-19) and *δ_C_* 169.05 (C-8) (Figure S3). According to the methodology, C-3, C-17 and C-25 possessed the same configuration with Compound **1**. As a result, the aglycone of Compound **3** was determined as 25(*R*)-spirost-6-one-7-en-3*β*,17*α*-diol. The aglycone of Compound **3** has never been reported. 

Acid hydrolysis of Compound **3** afforded d-glucose and l-rhamnose in accordance with the anomeric proton signals at *δ_H_* 4.52 (d, *J* = 7.75 Hz) and *δ_H_* 5.22 (br s) as well as the anomeric carbon signals at *δ_C_* 100.89 and *δ_C_* 102.16. Compared to **1** and **2**, Compound **3** also obtained the sugar chain Rha-(1→4)-Glc-, as was proven by HMBC spectrum (Figure S3). Thus, Compound **3** was elucidated as 25(*R*)-spirost-6-one-7-en-3*β*,17*α*-diol-3-*O*-*α*-l-rhamnopyranosyl-(1→2)-*β*-d-glucopyranoside, named Pavitnoside C (**3**).

Pavitnoside D (**4**), white amorphous solid, was positive to Liebermann–Burchard and Molisch chemical reactions. The molecular formula was established as C_39_H_60_O_14_ from the [M + Na]^+^ ion at *m*/*z* 775.3849 [M + Na]^+^ (calcd. for C_39_H_60_O_14_Na, 775.3881) in the HR-ESI-MS and the ^13^C-NMR spectral data. Compared to **3**, Compound **4** exactly possessed the same structure of aglycone and the monosaccharides experimented through acid hydrolysis. The main distinction was the sugar sequencing linkages depended on the correlation between *δ_H_* 5.21 (Rha H-1) and *δ_C_* 79.43 (Glc C-4). Thus, Compound **4** was established as 25(*R*)-spirost-6-one-7-en-3*β*,17*α*-diol-3-*O*-*α*-l-rhamnopyranosyl-(1→4)-*β*-d-glucopyranosie, named Pavitnoside D (**4**).

The six known steroidal saponins **5**–**10** were determined as 25(*R*)-spirost-5-en-3*β*,17*α*-diol-3-*O*-*α*-l-rhamnopyranosyl-(1→2)-*β*-d-glucopyranoside (**5**) [26], 25(*S*)-spirost-5-en-3*β*,17*α*-diol-3-*O*-*α*-l-rhamnopyranosyl-(1→2)-*β*-d-glucopyranoside (**6**) [27], 25(*R*)-spirost-5-en-3*β*,17*α*-diol-3-*O*-*α*-l-rha mnopyranosyl-(1→3)-*β*-d-glucopyrano side (**7**) [28], 25(*R*)-diosgenin-3-*O*-*α*-l-rhamnopyranosyl-(1→2)-*α*-l-rhamnopyranosyl-(1→3)-*β*-d-glucopyranoside (**8**) [29], 25(*R*)-spirost-5-en-3*β*,17*α*-diol-3-*O*-*α*-l-rhamnopyranosyl-(1→4)-[*α*-l-rhamnopyranosyl-(1→2)]-*β*-d-glucopyranoside (**9**) [30], and 25(*R*)-diosgenin-3-*O*-*α*-l-rhamnopyranosyl-(1→2)-*β-*d-glucopyranoside (**10**) [31] by comparison of the physical and spectroscopic data (Supplementary Materials, Tables S1 and S2) with literature values.

Since steroidal saponins have been reported to possess varying cytotoxic activity against various cancer cell lines [32,33], human glioblastoma U87MG and U251 cells were selected to evaluate the cytotoxicities of the obtained spirostanol glycosides **1**–**10**. The results of their cytotoxicities are shown in Table 2. Compounds **8** and **9** characterized at a branched trisaccharide chain attached to C-3 of the aglycone, exhibited significant cytotoxicities against the two test tumor cells, while saponin **1** displayed weak anti-proliferative activity on U87MG cell line. Saponins **2**–**7** and **10** possessing a disaccharide chain at C-3 of the aglycone displayed almost no activity (IC_50_ > 100 μM). It is worth noting that Compounds **1**–**10** did not affect the growth of primary cultures of human astrocytes. In fact, exposure of the astrocytes to the highest concentrations of **1**–**10** (100 μM) for 72 h did not result in any statistically significant change in cell viability, with the inhibition ranging from 3.8% to 9.1% (*p* > 0.05). Meanwhile, the viability of astrocytes treated with 100 μM ACNU for 72 h decreased to 67.4% (*p* < 0.05). Comparing **1** with **2**–**7**, the acetyl group may influence the cytotoxic activity. On the other hand, it seemed that greater number of sugar residues at C-3 of the aglycone showed higher cytotoxic activity. However, the structure–activity relationship (SAR) for the antitumor activity of saponins involved the type of the aglycones; the type, number, sequence and binding sites of the sugar moieties; etc. [1]. For the limitation in the number of the isolated steroidal saponins, further studies on the cytotoxicity of the related saponins from *P. vietnamensis* are necessary to clarify their SAR.

## 3. Experimental Section

### 3.1. General

Optical rotations were measured on a Perkin-Elemr 241 MC digital polarimeter (German Perkin-Elmer Corporation, Boelingen, Germany). 1D and 2D-NMR spectral experiments were measured in CD_3_OD on a Bruker AVANCE-500 spectrometer (Bruker Corporation, Karlsruhe, Germany), with TMS as internal standard. IR spectra were recorded on a Shimadzu IRPrestige-21 spectrophotometer (Shimadzu Corporation, Tokyo, Japan). ESI-MS and HR-ESI-MS spectra were carried out on a Waters Quattro Micromass mass spectrometer (Waters, Shanghai, China). Column chromatographies (CC) were performed on silica gel H (10−40 μm, Qingdao Marine Chemical Inc., Qingdao, China), ODS Silica gel (Lichroprep RP-18, 40-63 μm, Merck Inc., Darmstadt, Germany) and Sephadex LH-20 (GE-Healthcare, Uppsala, Sweden). GC analysis was performed on an Agilent 6890N apparatus using an HP-5 capillary column (30 m × 0.32 mm, 0.25 μm) and an FID detector with an initial temperature of 120 °C for 2 min and then temperature programming to 280 °C at the rate of 10 °C/min. HPLC were carried out on a Dionex P680 liquid chromatograph equipped with a UV 170 UV/Vis detector at 206 nm and 225 nm using a YMC-Pack ODS-A column (250 × 20 mm. D, S-5 μm, 12 nm, YMC, Kyoto, Japan) for semi-preparation. TLC detections were carried out on pre-coated plates with RP-18 (Merck) and silica GF254 (Qingdao Marine Chemical Inc., Qingdao, China) with 20% H_2_SO_4_ followed by heating for three minutes. Standards for d-glucose (D-Glc) and l-rhamnose (L-Rha) were purchased from Sigma Chemical Co. (St. Louis, MO, USA).

### 3.2. Plant Material

The dried rhizomes of *Paris vietnamensis* were collected from Ya’an, Sichuan Province, China in May 2016, and were identified by one of the authors, Haifeng Tang. The voucher sample (No. 20160520) was deposited in the Herbarium of Institute of Materia Medica, School of Pharmacy, Fourth Military Medical University, Xi’an, China.

### 3.3. Extraction and Isolation

The dried rhizomes of *Paris vietnamensis* (2 kg) were cut into pieces and refluxed with 70% ethanol (10 L) thrice (each 1.5 h). The solution was merged and condensed with a vacuum rotary evaporator to receive a syrupy residue (630 g). The extraction was suspended in water (3 L) and successively extracted with petroleum ether and water saturated *n*-BuOH. The water saturated *n*-BuOH layer was evaporated under reduced pressure to afford an extraction (200 g). The crude extract was offered to silica gel column chromatography (CC) and eluted by gradient eluent of CHCl_3_–MeOH–H_2_O (100:1:0, 50:1:0, 20:1:0, 15:1:0, 8:1:0.1, 8:2:0.2, 7:2.5:0.25, 6.5:3.5:0.1, 6:3:0.3) to get 15 fractions (Fr.1–15) depending on the TLC results. Fr.9 (2.0 g) was eluted by CHCl_3_–MeOH (1:1) on Sephadex LH-20 to get rid of pigmentum and then separated into two yields, Fr.9-1 (100 mg) and Fr.9-2 (600 mg), on ODS silica gel. Fr.9-2 was further isolated by semi-preparative HPLC using MeCN–H_2_O (65:35) as the mobile phase at a flow rate of 6.0 mL/min to afford Compounds **1** (4.6 mg, *t_R_* = 22.9 min), **2** (3.2 mg, *t_R_* = 15.9 min) and **5** (2.4 mg, *t_R_* = 13.5 min). Fr.11 (17.6 g) was eluted by MeOH on Sephadex LH-20 to get rid of pigmentum and was subjected to silica gel column chromatography (CC) and eluted by gradient eluent of CHCl_3_–MeOH–H_2_O (10:1:0.1, 8:1:0.1, 6:3:0.3) to produce Fr.11-1 (9.2 g) and Fr.11-2 (500 mg). Fr.11-1 was further separated on ODS silica gel to give five fractions. Then, Fr.11-1-2 (86 mg) was isolated by semi-preparative HPLC using MeCN–H_2_O (50:50) as the mobile phase at a flow rate of 8.0 mL/min to afford Compounds **3** (4.0 mg, *t_R_* = 8.2 min), **6** (83.4 mg, *t_R_* = 16.7 min) and **7** (3.3 mg, *t_R_* = 32.9 min). Fr.12 (24.0 g) was eluted by CHCl_3_–MeOH (1:1) on Sephadex LH-20 to get rid of pigmentum and was subjected to silica gel column chromatography (CC) and eluted by gradient eluent of CHCl_3_–MeOH–H_2_O (20:1:0.1, 10:1:0, 8:2:0.2, 7:2.5:0.25) to produce three fractions. Fr.12-1 (1.2 g) was isolated by semi-preparative HPLC using MeCN–H_2_O (80:20) as the mobile phase at a flow rate of 8.0 mL/min to afford Compounds **4** (3.8 mg, *t_R_* = 13.3 min) and **8** (5.4 mg, *t_R_* = 9.4 min). Fr.12-2 (4.2 g) was isolated by semi-preparative HPLC using MeCN–H_2_O (70:30) as the mobile phase at a flow rate of 8.0 mL/min to afford Compound **9** (16.8 mg, *t_R_* = 19.5 min) and Compound **10** (11.2 mg, *t_R_* = 18.7 min). The purity of all compounds was assessed by HPLC as more than 95%.

### 3.4. Compound Characterization Data

*Pavitnoside A* (**1**): Yellowish amorphous solid, ${\left[\alpha \right]}_{D}^{22}$ −25.1 (*c* 0.01, MeOH); IR (KBr) *ν_max_* (cm^−1^): 3422, 2933, 1422, 1056, 870; Positive ESI-MS *m*/*z* 803.31 [M + Na]^+^; Negative ESI-MS *m*/*z* 779.25 [M − H]^−^, 737.26 [M − 43]^−^, 591.21 [M − 146 − 43]^−^; Positive HR-ESI-MS *m*/*z* 803.4170 [M + Na]^+^; ^1^H-NMR (800 MHz, CD_3_OD) and ^13^C-NMR (201 MHz, CD3OD) data, see Table 1.

*Pavitnoside B* (**2**): White amorphous solid, ${\left[\alpha \right]}_{D}^{22}$ −49.2 (*c* 0.05, MeOH); IR (KBr) *ν_max_* (cm^−1^): 3420, 2943, 1661, 1070, 989, 892; Positive ESI-MS *m*/*z* 777.47 [M + Na]^+^; Negative ESI-MS *m*/*z* 753.28 [M − H]^−^, 737.29 [M − 17]^−^; Positive HR-ESI-MS *m*/*z* 777.4044 [M + Na]^+^; ^1^H-NMR (800 MHz, CD_3_OD) and ^13^C-NMR (201 MHz, CD_3_OD) data, see Table 1.

*Pavitnoside C* (**3**): White amorphous solid, ${\left[\alpha \right]}_{D}^{22}$ −92.2 (*c* 0.06, MeOH); IR (KBr) *ν_max_* (cm^−1^): 3430, 2920, 1665, 1159, 1070, 989, 920, 900; Positive ESI-MS *m*/*z* 775.43 [M + Na]^+^, 1527.94 [2M + Na]^+^, 445.37 [M − 145 − 162]^+^, 427.35 [M − 146 − 162 − 17]^+^, 409.35 [M − 146 − 162 − 17 − H_2_O]^+^; Positive HR-ESI-MS *m*/*z* 775.3884 [M + Na]^+^; ^1^H-NMR (500 MHz, CD_3_OD) and ^13^C-NMR (125 MHz, CD_3_OD) data, see Table 1.

*Pavitnoside D* (**4**): White amorphous solid, ${\left[\alpha \right]}_{D}^{22}$ −93.1 (*c* 0.06, MeOH); IR (KBr) *ν_max_* (cm^−1^): 3429, 2930, 1660, 1160, 1050, 989, 900; Positive HR-ESI-MS *m*/*z* 775.3849 [M + Na]^+^; ^1^H-NMR (500 MHz, CD_3_OD) and ^13^C-NMR (125 MHz, CD_3_OD) data, see Table 1.

### 3.5. Acid Hydrolysis and GC Analysis of the Sugar Moieties in ***1**–**4***

Compounds **1**–**4** (each 2 mg) were heated in an ampule with 2 mol/L CF_3_COOH (2 mL) at 120 °C for 2 h. The reaction mixture was extracted with CHCl_3_ (3 × 5 mL). The aqueous layer was concentrated in vacuo; 1 mL pyridine and 2 mg NH_2_OH·HCl were added to the dried residue; and the mixture was stirred at 90 °C for 1 h. After the reaction mixtures were cooled, 1.5 mL of Ac_2_O was added and the mixtures were heated at 90 °C for 1 h. The reaction mixtures were concentrated under reduced pressure, and the resulting aldononitrile peracetates were analyzed by GC. The carbohydrates were determined by comparing the retention times with standard aldononitrile peracetates prepared from authentic sugars by the same procedure performed for the sample [1,34]. Retention times for authentic sugars after being derivatized were 5.61 min (L-Rha) and 11.56 min (D-Glc), respectively. L-Rha and D-Glc were identified in a ratio of 1:1 for Compounds **1**–**4**.

### 3.6. Cytotoxicity Assay for Compounds ***1**–**10***

Human glioma cell lines U251 and U87MG were obtained from the Cell Bank of Chinese Academy of Science (Shanghai, China). Cultured primary astrocytes were obtained from a slightly impaired brain tissue fragment of a volunteer with cerebral trauma who consented to the procedure as described previously [35]. Acquisition of the tissue was approved by the local medical research ethics committee at Xijing Hospital, Fourth Military Medical University. The cell lines were cultured in DMEM (Corning, Beijing, China) supplemented with 10% FBS (Ausbian, Harbin, China) at 37 °C with 5% CO_2_. The logarithmic phase cells were seeded on 96-well plates at the concentration of 5000 cells/well and incubated with various concentrations (100, 10, 1, 0.1, and 0.01 μM in medium containing less than 0.1% DMSO) of Compounds **1**–**10** in triple wells for 72 h. Nimustine hydrochloride (ACNU, Sigma, ≥ 99%, Shanghai, China) was used as the positive control. Cell viability was determined according to reported assay methods using commercial CCK8 kit (7sea biotech, Shanghai, China) [36,37]. The optical density of each well was measured with a Bio-Rad 680 microplate reader at 450 nm (Bio-Rad Corporation, Hercules, CA, USA). Cytotoxicity was expressed as the concentration of drug inhibiting cell growth by 50% (IC_50_).

### 3.7. Statistical Analysis

All data are presented as the mean ± standard deviation (SD) and analyzed using SPSS version 19.0 (SPSS Inc., Chicago, IL, USA). Unpaired test and one-way Analysis of Variance (ANOVA) followed by LSD test were performed for the differences between different groups. A value of *p* < 0.05 was considered as statistically significant difference.

## 4. Conclusions

Phytochemical investigation of *P. vietnamensis* afforded 10 compounds, including four new compounds. The aglycones of Compounds **2** and **3** had never been isolated in the phytochemical studies and the ^1^H-NMR and ^13^C-NMR spectroscopic data were given for the first time in this study.

The experiments of cytotoxicity of all compounds showed that **8** and **9** exhibited notable cytotoxicity against the U87MG and U251 cell lines. However, Compounds **1**–**10** did not affect the growth of primary cultures of human astrocytes. It is suggested that saponins **8** and **9** are reliable candidates for chemotherapeutic treatment of human glioma.

## Figures and Tables

**Table:** 

	Aglycone	R_1_	R_2_	R_3_	R_4_	Configuration of C-25
**1**	I	S_1_	COCH_3_	OH	CH_3_	*25R*
**2**	I	S_1_	H	OH	CH_2_OH	*25R*
**3**	II	S_1_	H	-	-	*25R*
**4**	II	S_2_	H	-	-	*25R*
**5**	I	S_1_	H	OH	CH_3_	*25R*
**6**	I	S_1_	H	OH	CH_3_	*25S*
**7**	I	S_3_	H	OH	CH_3_	*25R*
**8**	I	S_4_	H	H	CH_3_	*25R*
**9**	I	S_5_	H	OH	CH_3_	*25R*
**10**	I	S_1_	H	H	CH_3_	*25R*

**Table 1:** ^1^H- and ^13^C-NMR data for Compounds **1–4** in CD_3_OD ^†^.

	1 ^a^		2 ^a^		3 ^b^		4 ^b^	
**1**	38.74	1.08 m, 1.90 m	38.75	1.09 m, 1.90 m	37.68	1.24 m, 2.01 m	37.69	1.21 m, 2.00 m
**2**	30.97	1.30 m, 1.59 m	30.91	1.29 m, 1.92 m	30.56	1.72 m, 2.05 m	30.57	1.72 m, 2.05 m
**3**	80.09	3.53 m	79.32	3.62 m	78.05	3.79 m	78.04	3.77 m
**4**	39.77	2.45 m, 2.30 t (11.1)	39.66	2.45 m, 2.30 t (11.2)	39.61	2.48 m, 2.73 m	39.61	2.46 m, 2.72 m
**5**	142.03	−	142.06	−	46.62	2.49 m	46.60	2.46 m
**6**	122.80	5.40 br s	122.75	5.39 br s	204.57	−	204.49	−
**7**	32.66	1.57 m, 1.70 m	33.83	1.64 m, 1.83 m	126.59	5.73 br s	126.59	5.73 br s
**8**	33.44	1.65 m	33.64	1.65 m	169.05	−	169.06	−
**9**	51.61	0.95 m	51.63	0.94 m	51.37	1.48 m	51.37	1.47 m
**10**	38.13	−	38.15	−	40.08	−	40.09	−
**11**	21.85	1.52 m, 1.62 m	21.70	1.51 m, 1.62 m	21.85	1.65 m	21.85	1.60 m, 1.70 m
**12**	33.00	1.59 m, 1.69 m	32.44	1.44 m, 1.58 m	32.67	1.53 m, 1.65 m	32.67	1.53 m, 1.70 m
**13**	46.00	−	46.25	−	46.66	−	46.66	−
**14**	54.07	1.72 m	53.87	1.71 m	47.27	2.03 m	47.27	2.00 m
**15**	32.24	1.27 m, 2.06 m	32.28	1.26 m, 2.06 m	34.22	1.39 m, 2.84 m	34.22	1.35 m, 2.80 m
**16**	90.68	4.01 m	90.08	4.08 dd (5.84, 7.68)	90.59	4.05 dd (6.45, 7.75)	90.58	4.04 dd (6.4, 7.76)
**17**	91.45	−	91.45	−	90.11	−	90.13	−
**18**	17.67	0.84 s	17.34	0.86 s	17.81	0.84 s	17.81	0.84 s
**19**	19.98	1.05 s	19.99	1.06 s	17.83	1.25 s	17.83	1.27 s
**20**	45.68	2.10 q (7.12)	53.15	2.25 dd (5.84, 8.56)	45.33	2.08 m	45.53	2.06 m
**21**	9.26	0.90 d (7.2)	59.55	3.61 m, 3.80 dd (8.64, 11.2)	9.45	0.90 d (7.20)	9.44	0.90 d (7.28)
**22**	110.10	−	110.17	−	111.11	−	111.10	−
**23**	33.35	1.58 m, 2.03 m	33.37	1.56 m, 2.02 m	32.31	1.32 m, 1.39 m	32.31	1.32 m, 1.39 m
**24**	29.59	1.45 m, 1.63 m	29.59	1.42 m, 1.60 m	29.56	1.46 m, 1.62 m	29.57	1.43 m, 1.61 m
**25**	31.44	1.61 m	31.37	1.61 m	31.42	1.61 m	31.43	1.60 m
**26**	67.85	3.34 m, 3.49 m	67.85	3.34 m, 3.47 m	67.82	3.35 m, 3.49 m	67.82	3.33 m, 3.48 m
**27**	17.64	0.80 d (6.4)	17.62	0.79 d (6.4)	17.64	0.86 d (6.3)	17.65	0.81 d (6.4)
**Glc**								
**1**	101.04	4.51 d (7.76)	100.66	4.48 d (7.76)	100.89	4.52 d (7.75)	100.87	4.53 d (7.76)
**2**	79.07	3.37 m	79.18	3.35 m	78.88	3.41 m	78.88	3.36 m
**3**	79.33	3.48 m	79.54	3.46 m	77.89	3.38 m	77.91	3.27 m
**4**	75.10	3.44 m	77.89	3.24 m	79.42	3.78 m	79.43	3.48 m
**5**	71.98	3.27 m	71.99	3.26 m	71.94	3.49 m	71.94	3.27 m
**6**	64.09	4.20 q (6.08), 4.35 dd (2.16, 11.92)	62.92	3.64 m, 3.85 m	62.90	3.65 m, 3.85 m	62.89	3.63 m, 3.83 m
**-COCH_3_**								
**1**	172.89	−						
**2**	20.89	2.05 s						
**Rha**								
**1**	102.33	5.20 br s	102.33	5.19 br s	102.16	5.22 br s	102.18	5.21 br s
**2**	72.36	3.91 m	72.38	3.91 m	72.30	3.92 m	72.31	3.92 m
**3**	72.52	3.66 m	72.53	3.66 m	72.51	3.66 m	72.51	3.65 m
**4**	74.09	3.40 m	74.09	3.39 m	74.07	3.40 m	74.06	3.38 m
**5**	69.91	4.12 dd (6.24, 9.60)	69.90	4.14 dd (6.24, 9.60)	69.85	4.12 dd (6.25, 9.50)	69.86	4.12 dd (6.24, 9.60)
**6**	18.12	1.26 d (6.24)	18.11	1.24 d (6.24)	18.13	1.26 d (6.3)	18.13	1.25 d (6.24)

^†^ Assignments aided by the DEPT, ^1^H-^1^H COSY, HSQC, HMBC, TOCSY, and NOESY experiments; ^a^ tested at 800 Hz, ^b^ tested at 500 Hz.

**Table 2:** Cytotoxicities of saponins **1**–**10** against two human cancer cell lines and primary cultured astrocytes in vitro (IC_50_, μM) ^a^.

Compounds	Astrocytes	Cell Lines	
		U251	U87MG
**1**	>100	>100	52.04 ± 1.28
**2**	>100	>100	>100
**3**	>100	>100	>100
**4**	>100	>100	>100
**5**	>100	>100	>100
**6**	>100	>100	>100
**7**	>100	>100	>100
**8**	>100	2.16 ± 0.65	2.33 ± 1.03
**9**	>100	3.14 ± 1.26	2.97 ± 0.94
**10**	>100	>100	>100
**ACNU ^b^**	>100	0.96 ± 0.05	0.88 ± 0.04

^a^ IC_50_ values are means from three independent experiments (average ± SD) in which each compound concentration was tested in three replicate wells; ^b^ Nimustine hydrochloride (ACNU) was the positive control.

## Supplementary Materials

The physical and spectroscopic data of saponins **5**–**10**, and NMR and MS spectra of saponins **1**–**4** are available online.

## References

[1] Liu Y., Tian X., Hua D., Cheng G., Wang K., Zhang L., Tang H., Wang M. (2016). New steroidal saponins from the rhizomes of *Paris delavayi* and their cytotoxicity. Fitoterapia.

[2] Wen F.Y., Yin H.X., Chen C., Liu X.B., Xue D., Chen T.Z., He J., Zhang H. (2012). Chemical characteristics of saponins from *Paris fargesii* var. *brevipetala* and cytotoxic activity of its main ingredient, paris saponin H. Fitoterapia.

[3] Watanabe S., Suzuki T., Hara F., Yasui T., Uga N., Naoe A. (2017). Polyphyllin D, a steroidal saponin in *Paris polyphylla*, induces apoptosis and necroptosis cell death of neuroblastoma cells. Pediatric Surg. Int..

[4] Zheng L., Zheng J., Zhao Y., Wang B., Wu L., Liang H. (2006). Three anti-tumor saponins from *Albizia julibrissin*. Bioorg. Med. Chem. Lett..

[5] Kawabata T., Cui M.Y., Hasegawa T., Takano F., Ohta T. (2011). Anti-inflammatory and anti-melanogenic steroidal saponin glycosides from Fenugreek (*Trigonella foenum-graecum* L.) seeds. Planta Medica.

[6] Deng D., Lauren D.R., Cooney J.M., Jensen D.J., Wurms K.V., Upritchard J.E., Cannon R.D., Wang M.Z., Li M.Z. (2008). Antifungal saponins from *Paris polyphylla Smith*. Planta Medica.

[7] Tian Y., Zheng L.H., Xu Z.Y., Sun L.Q., Gao C.K., Zheng Q.Z., Zhang Z.H., Shu Y. (1986). Clinical and pharmacological study of the hemostatic action of *Rhizoma Paridis* by contraction of uterus. J. Tradit. Chin. Med..

[8] Zaki A.A., Ali Z., Wang Y.H., El-Amier Y.A., Khan S.I., Khan I.A. (2017). Cytotoxic steroidal saponins from *Panicum turgidum* Forssk. Steroids.

[9] Jing S.S., Wang Y., Li X., Man S.L., Gao W.Y. (2017). Chemical constituents and antitumor activity from *Paris polyphylla Smith* var. *yunnanensis*. Nat. Prod. Res..

[10] Yang B.Y., Bi X.Y., Liu Y., Li G.Y., Yin X., Kuang H.X. (2017). Four New Glycosides from the Rhizoma of *Anemarrhena asphodeloides*. Molecules.

[11] Pereira G.M., Ribeiro M.G., da Silva B.P., Parente J.P. (2017). Structural characterization of a new steroidal saponin from *Agave angustifolia* var. *Marginata* and a preliminary investigation of its in vivo antiulcerogenic activity and in vitro membrane permeability property. Bioorg. Med. Chem. Lett..

[12] Li H. (1998). The Genus Paris (Trilliaceae).

[13] Huang Y., Cui L.J., Wang Q., Ye W.C. (2006). Separation and identification of active constituents of *Paris vietnamensis*. Acta Pharm. Sin..

[14] Chen P.Y., Chen C.H., Kuo C.C., Lee T.H., Kuo Y.H., Lee C.K. (2011). Cytotoxic steroidal saponins from *Agave sisalana*. Planta Medica.

[15] Liu H., Chou G.X., Wang J.M., Ji L.L., Wang Z.T. (2011). Steroidal saponins from the rhizomes of *Dioscorea bulbifera* and their cytotoxic activity. Planta Medica.

[16] Zhao Y., Kang L.P., Liu Y.X., Liang Y.G., Tan D.W., Yu Z.Y., Cong Y.W., Ma B.P. (2009). Steroidal saponins from the rhizome of *Paris polyphylla* and their cytotoxic activities. Planta Medica.

[17] Agrawal P.K., Jain D.C., Pathak A.K. (1995). NMR spectroscopy of steroidal sapogenins and steroidal saponins: An update. Magn. Reson. Chem..

[18] Agrawal P.K., Jain D.C., Gupta R.K., Thakur R.S. (1985). Carbon-13 NMR spectroscopy of steroidal sapogenins and steroidal saponins. Phytochemistry.

[19] Xiao C.M., Huang J., Zhong X.M., Tan X.Y., Deng P.C. (2010). Two New Homo-aro-cholestane Glycosides and a New Cholestane Glycoside from the Roots and Rhizomes of *Paris polyphylla* var. *Pseudothibetica*. Helv. Chim. Acta.

[20] Agrawal P.K. (2005). Assigning stereodiversity of the 27-Me group of furostane-type steroidal saponins via NMR chemical shifts. Steroids.

[21] Zhang J., Ma B.P., Kang L.P., Yu H.S., Yang Y., Yan X.Z. (2006). NMR studies of two furostanol saponins isolated from *Polygonatum Kingianum*. Chin. J. Magn. Reson..

[22] Mahato S.B., Sahu N.P., Ganguly A.N. (1981). Steroidal saponins from *Dioscorea floribunda*: Structures of floribundasaponins A and B. Phytochemistry.

[23] Tian X., Feng J., Tang H., Zhao M., Li Y., Hai W., Zhang X. (2013). New cytotoxic triterpenoid saponins from the whole plant of *Clematis lasiandra Maxim*. Fitoterapia.

[24] Kasai R., Okihara M., Asakawa J., Mizutani K., Tanaka O. (1979). ^13^C NMR study of *α*-anomeric and *β*-anomeric pairs of d-mannopyranosides and l-rhamnopyranosides. Tetrahedron.

[25] Zhang Y., Yang C.R., Zhang Y.J. (2016). Steroidal saponins from the rhizomes of *Polygonatum prattii*. J. Asian Nat. Prod. Res..

[26] Xie B.B., Liu H.Y., Ni W., Chen C.X. (2009). Ypsilandrosides C-G, five new spirostanol saponins from *Ypsilandra thibetica*. Steroids.

[27] Kang L.P., Huang Y.Y., Zhan Z.L., Liu D.H., Peng H.S., Nan T.G., Zhang Y., Hao Q.X., Tang J.F., Zhu S.D. (2017). Structural characterization and discrimination of the *Paris polyphylla* var. *yunnanensis* and *Paris vietnamensis* based on metabolite profiling analysis. J. Pharm. Biomed. Anal..

[28] Tang L.Y., Wang Z.J., Wu H.W., Yokosuka A., Mimaki Y. (2014). Steroidal glycosides from the underground parts of *Dracaena thalioides* and their cytotoxic activity. Phytochemistry.

[29] Khodakov G.V., Akimov Y.A., Shashkov A.S., Kintia P.K., Grishkovets V.I. (1996). Triterpene and steroid saponins isolated from two *Melilotus* species. Oxyg. Transp. Tissue XXXIII.

[30] Li Y.H., Liu C.X., Xiao D., Han J., Yue Z.G., Sun Y., Fan L., Zhang F., Meng J., Zhang R. (2015). *Trillium tschonoskii* steroidal saponins suppress the growth of colorectal Cancer cells in vitro and in vivo. J. Ethnopharmacol..

[31] Lee H.J., Watanabe B., Nakayasu M., Onjo M., Sugimoto Y., Mizutani M. (2017). Novel steroidal saponins from *Dioscorea esculenta* (Togedokoro). Biosci. Biotechnol. Biochem..

[32] Lu Y.Y., Luo J.G., Huang X.F., Kong L.Y. (2009). Four new steroidal glycosides from *Solanum torvum* and their cytotoxic activities. Steroids.

[33] Tian X.R., Tang H.F., Lin H.W., Cheng G., Wang S.W., Zhang X. (2013). Saponins: The potential chemotherapeutic angents in pursuing new anti-glioblastoma drugs. Mini-Rev. Med. Chem..

[34] Fu Q., Zan K., Zhao M., Zhou S., Shi S., Jiang Y., Tu P. (2010). Triterpene saponins from *Clematis chinensis* and their potential anti-inflammatory activity. J. Nat. Prod..

[35] Lin H., Zhang X., Cheng G., Tang H.F., Zhang W., Zhen H.N., Cheng J.X., Liu B.L., Cao W.D., Dong W.P. (2008). Apoptosis induced by ardipusilloside III through BAD dephosphorylation and cleavage in human glioblastoma U251MG cells. Apoptosis.

[36] Dang Y., Wu W., Xu Y., Mu Y., Xu K., Wu H., Zhu Y., Zhang C. (2015). Effects of low-level laser irradiation on proliferation and functional protein expression in human RPE cells. Lasers Med. Sci..

[37] Wang Z.Y., Yang J., Xu G., Wang W., Liu C.H., Yang H.H., Yu Z.B., Lei Q.Q., Xiao L., Xiong J. (2014). Targeting miR-381-NEFL axis sensitizes glioblastoma cells to temozolomide by regulating stemness factors and multidrug resistance factors. Oncotarget.

