# Supplementary material for: New Steroidal Saponins from the Rhizomes of Paris vietnamensis and Their Cytotoxicity
Source: Molecules. 2018 Mar 6;23(3):588. doi: 10.3390/molecules23030588 (PMC6017139; doi:10.3390/molecules23030588)
Supplement: Supplementary file 1 [file molecules-23-00588-s001.pdf]

Supporting Information

New steroidal saponins from the rhizomes of *Paris vietnamensis* and their cytotoxicity

Yang Liu [1, †], Minchang Wang [2, 3, †], Ke Liu [2,3], Pengcheng Qiu [1], Shan Zhang [1], Yunyang Lu [1], Na Tang [1], Haifeng Tang [1,*]

[1] Institute of Materia Medica, School of Pharmacy, Fourth Military Medical University, Xi'an 710032, PR China; so870823@163.com (Y.L.); tanghaifeng71@163.com (H.-F.T.)

[2] State Key Laboratory of Fluorine & Nitrogen Chemicals, Xi'an 710065, PR China; wmc204@163.com (M.-C.W.); happycoco5133@163.com (K. L.)

[3] Xi'an Modern Chemistry Research Institute, Xi'an 710065, PR China; wmc204@163.com (M.-C.W.); happycoco5133@163.com (K. L.)

* Correspondence: tanghaifeng71@163.com (H.-F.T.); Tel.: +86-29-8477-4748 (H.-F.T.)

† The authors contribute equally to this work.

Contents

Figure S1 Structures of compounds **1–10** from *Paris vietnamensis*

Figure S2 Key ^{1}H-^{1}H COSY, HMBC and NOESY correlations of compound **2**.

Figure S3 Key ^{1}H-^{1}H COSY, HMBC and NOESY correlations of compound **3**.

Figure S4 Key ^{1}H-^{1}H COSY, HMBC and NOESY correlations of compound **4**

Physical and spectroscopic data of compounds **5**–**10**

Table S1 ^{13}C-NMR data of the aglycone moieties of compounds **5-10**

Table S2 ^{13}C-NMR data of the sugar portion of compounds **5-10**

NMR (1D and 2D) and MS spectra of compounds 1–4

S1. Positive HR-ESI-MS spectrum of compound **1**

S2. Positive and Negative ESI-MS spectrum of compound **1**

S3. ^{1}H NMR (800 MHz, CD_3OD) spectrum of compound **1**

S4. ^{13}C NMR (201 MHz, CD_3OD) spectrum of compound **1**

S5. DEPT 135 spectrum of compound **1**

S6. HSQC spectrum of compound **1**

S7. HMBC spectrum of compound **1**

S8. ^{1}H-^{1}H COSY spectrum of compound **1**

S9. NOESY spectrum of compound **1**

S10. TOCSY spectrum of compound **1**

S11. Positive HR-ESI-MS spectrum of compound **2**

S12. Positive and Negative ESI-MS spectrum of compound **2**

S13. ^{1}H NMR (800 MHz, CD_3OD) spectrum of compound **2**

S14. ^{13}C NMR (201 MHz, CD_3OD) spectrum of compound **2**

S15. DEPT 135 spectrum of compound **2**

S16. HSQC spectrum of compound **2**

S17. HMBC spectrum of compound **2**

S18. ^{1}H-^{1}H COSY spectrum of compound **2**

S19. NOESY spectrum of compound **2**

S20. TOCSY spectrum of compound **2**

S21. Positive HR-ESI-MS spectrum of compound **3**

S22. Positive ESI-MS spectrum of compound **3**

S23. ^{1}H NMR (500 MHz, CD_3OD) spectrum of compound **3**

S24. ^{13}C NMR (125 MHz, CD_3OD) spectrum of compound **3**

S25. DEPT 135 spectrum of compound **3**

S26. HSQC spectrum of compound **3**

S27. HMBC spectrum of compound **3**

S28. ^{1}H-^{1}H COSY spectrum of compound **3**

S29. NOESY spectrum of compound **3**

S30. TOCSY spectrum of compound **3**

S31. Positive HR-ESI-MS spectrum of compound **4**

S32. ^{1}H NMR (500 MHz, CD_3OD) spectrum of compound **4**

S33. ^{13}C NMR (125 MHz, CD_3OD) spectrum of compound **4**

S34. DEPT 135 spectrum of compound **4**

S35. HSQC spectrum of compound **4**

S36. HMBC spectrum of compound **4**

S37. ^{1}H-^{1}H COSY spectrum of compound **4**

S38. NOESY spectrum of compound **4**

S39. TOCSY spectrum of compound **4**

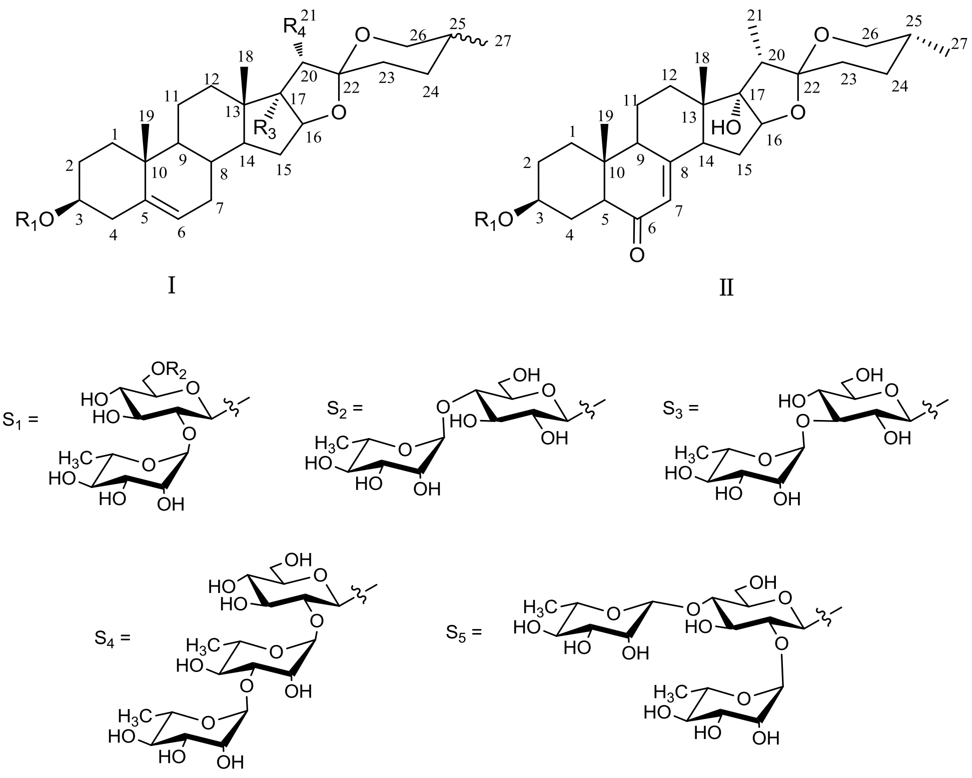

	Aglycone	R_1	R_2	R_3	R_4	Configuration of C-25
**1**	I	S_1	$COCH_3$	OH	CH_3	*25R*
**2**	I	S_1	H	OH	CH_2OH	*25R*
**3**	II	S_1	H	-	-	*25R*
**4**	II	S_2	H	-	-	*25R*
**5**	I	S_1	H	OH	CH_3	*25R*
**6**	I	S_1	H	OH	CH_3	*25S*
**7**	I	S_3	H	OH	CH_3	*25R*
**8**	I	S_4	H	H	CH_3	*25R*
**9**	I	S_5	H	OH	CH_3	*25R*
**10**	I	S_1	H	H	CH_3	*25R*

Figure S1 Structures of compounds **1–10** from *Paris vietnamensis*

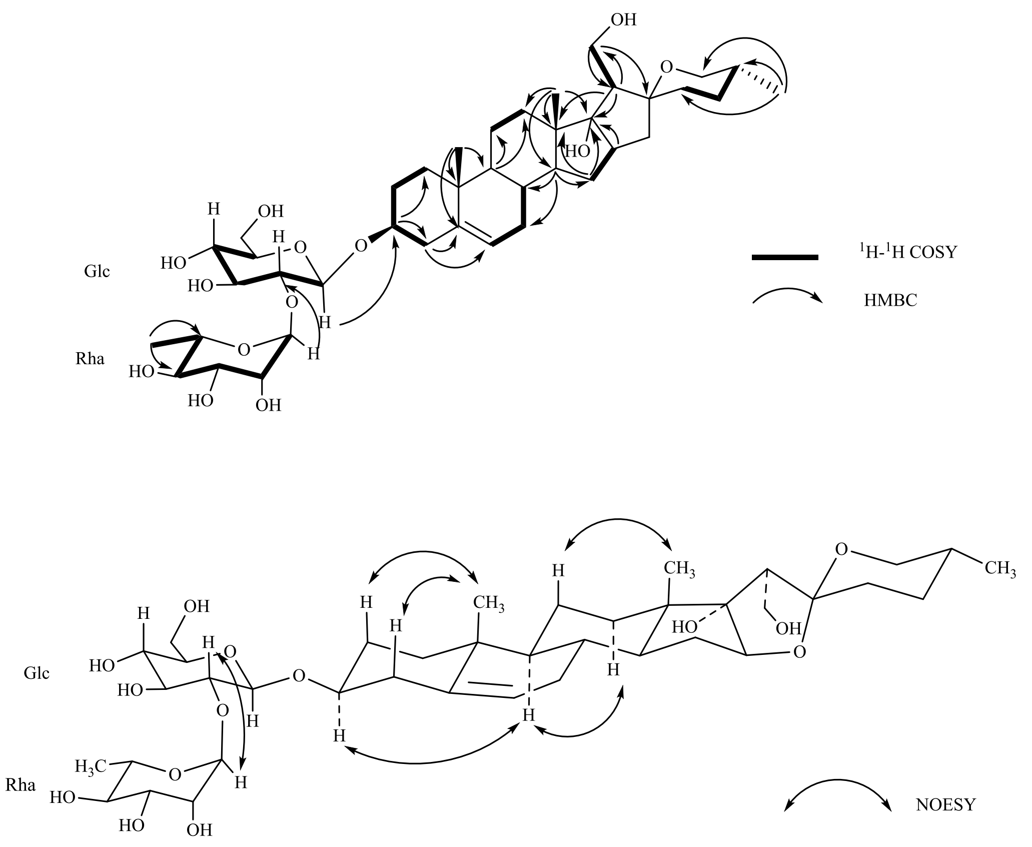

Figure S2 Key ^{1}H-^{1}H COSY, HMBC and NOESY correlations of compound **2**

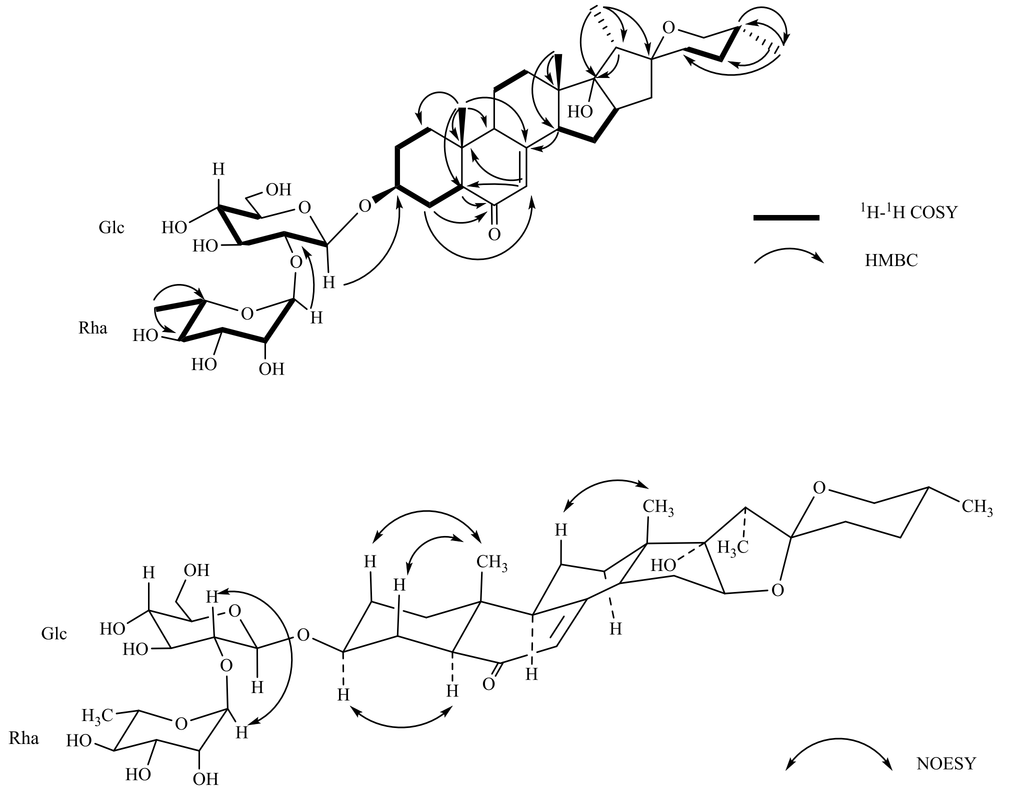

Figure S3 Key ^{1}H-^{1}H COSY, HMBC and NOESY correlations of compound **3**

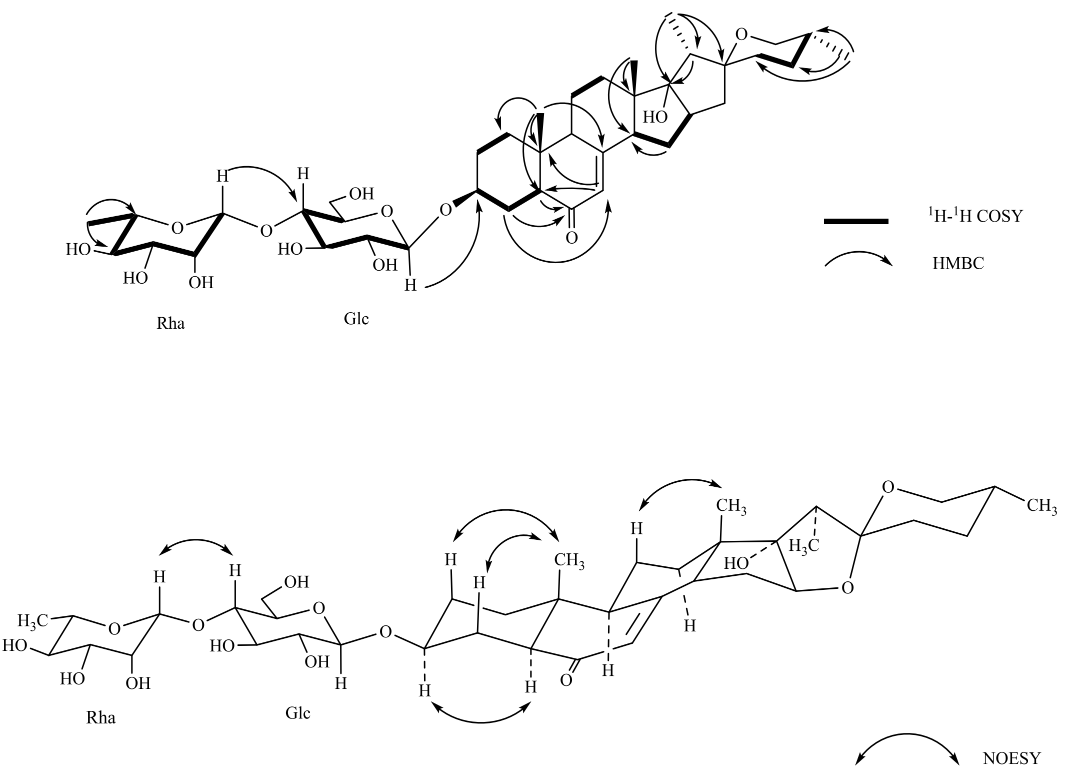

Figure S4 Key ^{1}H-^{1}H COSY, HMBC and NOESY correlations of compound **4**

Physical and spectroscopic data of compounds 5–10

Compound **5**: White amorphous powder; ^{1}H-NMR (500MHz, CD_3OD) δ_H: 0.83 (3H, *s*, H-18), 1.05 (3H, *s*, H-19), 0.89 (3H, *d, J* = 6.4 Hz, H-21), 0.80 (3H, *d, J* = 7.2 Hz, H-27), 5.39 (1H, *br s*, H-6), 1.26 (3H, *d, J* = 6.24 Hz, Me of Rha I), 4.48 (1H, *d, J* = 7.76 Hz, Glc H-1), 5.20 (1H, *br s*, Rha H-1); ^{13}C-NMR data, see **Table S1** and **Table S2**; ESI-MS (negative ion mode) 737 $[M - H]^-$. Compared to the physical and spectroscopic data with literature values, compound **5** was identified as 25(*R*)-spirost-5-en-3*β*,17*α*-diol-3-*O*-*α*-L-rhamnopyranosyl- (1 → 2)-*β*-D-glucopyranoside [1].

Compound **6**: White amorphous powder; ^{1}H-NMR (500MHz, CD_3OD) δ_H: 0.89 (3H, *s*, H-18), 1.06 (3H, *s*, H-19), 0.93 (3H, *d, J* = 7.44 Hz, H-21), 1.09 (3H, *d, J* = 7.12 Hz, H-27), 5.39 (1H, *br s*, H-6), 1.24 (3H, *d, J* = 6.24 Hz, Me of Rha I), 4.48 (1H, *d, J* = 7.76 Hz, Glc

H-1), 5.19 (1H, *br s*, Rha H-1); ^{13}C-NMR data, see **Table S1** and **Table S2**; ESI-MS (negative ion mode) 737 [M – H]$^{-}$. Compared to the physical and spectroscopic data with literature values, compound **6** was identified as 25(*S*)-spirost-5-en-3*β*,17*α*-diol-3-*O*-*α*-L-rhamnopyranosyl -(1 → 2) -*β*-D-glucopyranoside [2].

Compound **7**: White amorphous powder; ^{1}H-NMR (500MHz, CD_3OD) δ_H: 0.83 (3H, *s*, H-18), 1.05 (3H, *s*, H-19), 0.89 (3H, *d*, *J* = 7.2 Hz, H-21), 0.80 (3H, *d*, *J* = 6.4 Hz, H-27), 5.39 (1H, *br s*, H-6), 1.24 (3H, *d*, *J* = 6.24 Hz, Me of Rha I), 4.48 (1H, *d*, *J* = 7.84 Hz, Glc H-1), 5.19 (1H, *br s*, Rha H-1); ^{13}C-NMR data, see **Table S1** and **Table S2**; ESI-MS (negative ion mode) 737 [M – H]$^{-}$. Compared to the physical and spectroscopic data with literature values, compound **7** was identified as 25(*R*)-spirost-5-en-3*β*,17*α*-diol-3-*O*-*α*-L-rhamnopyranosyl-(1 → 3)-*β*-D-glucopyranoside [3].

Compound **8**: White amorphous powder; ^{1}H-NMR (500MHz, CD_3OD) δ_H: 0.82 (3H, *s*, H-18), 1.07 (3H, *s*, H-19), 0.98 (3H, *d*, *J* = 7.0 Hz, H-21), 0.81 (3H, *d*, *J* = 6.4 Hz, H-27), 5.40 (1H, *br s*, H-6), 1.27 (3H, *d*, *J* = 6.25 Hz, Me of Rha I), 1.26 (3H, *d*, *J* = 6.25 Hz, Me of Rha II), 4.51 (1H, *d*, *J* = 7.8 Hz, Glc H-1), 5.22 (1H, *br s*, Rha I H-1), 4.85 (1H, *br s*, Rha II H-1); ^{13}C-NMR data, see **Table S1** and **Table S2**; ESI-MS (negative ion mode) 867 [M – H]$^{-}$. Compared to the physical and spectroscopic data with literature values, compound **8** was identified as 25(*R*)-diosgenin-3-*O*-*α*-L-rhamnopyranosyl-(1 → 2)-*α*-L-rhamnopyranosyl- (1→3)-*β*-D-glucopyranoside [4].

Compound **9**: White amorphous powder; ^{1}H-NMR (500MHz, CD_3OD) δ_H: 0.84 (3H, *s*,

H-18), 1.06 (3H, *s*, Me-19), 0.90 (3H, *d*, *J* = 7.25 Hz, H-21), 0.80 (3H, *d*, *J* = 6.3 Hz, H-27), 5.40 (1H, *br s*, H-6), 1.25 (3H, *d*, *J* = 6.25 Hz, Me of Rha I), 1.27 (3H, *d*, *J* = 6.25 Hz, Me of Rha II), 4.51 (1H, *d*, *J* = 7.85 Hz, Glc H-1), 5.21 (1H, *br s*, Rha I H-1), 4.85 (1H, *br s*, Rha II H-1); ^{13}C-NMR data, see **Table S1** and **Table S2**; ESI-MS (negative ion mode) 883 [M – H]$^-$. Compared to the physical and spectroscopic data with literature values, compound **9** was identified as 25(*R*)-spirost-5-en-3*β*,17*α*-diol-3-*O*-*α*-L-rhamnopyranosyl-(1 → 4)-[*α*-L-rhamnopyranosyl-(1→2)]-*β*-D-glucopyranoside [5].

Compound **10**: White amorphous powder; ^{1}H-NMR (500MHz, CD_3OD) δ_H: 0.81 (3H, *s*, H-18), 1.05 (3H, *s*, Me-19), 0.96 (3H, *d*, *J* = 7.0 Hz, H-21), 0.79 (3H, *d*, *J* = 6.4 Hz, H-27), 5.38 (1H, *br s*, H-6), 1.24 (3H, *d*, *J* = 6.25 Hz, Me of Rha), 4.48 (1H, *d*, *J* = 7.75 Hz, Glc H-1), 5.19 (1H, *br s*, Rha H-1); ^{13}C-NMR data, see **Table S1** and **Table S2**; ESI-MS (negative ion mode) 721 [M – H]$^-$. Compared to the physical and spectroscopic data with literature values, compound **10** was identified as 25(*R*)-diosgenin-3-*O*-*α*-L-rhamnopyranosyl-(1→2)-*β*-D- glucopyranoside [6].

Table S1 **^{13}C-NMR data of the aglycone moieties of compounds 5-10**

Position	Compounds					
	**5**	**6**	**7**	**8**	**9**	**10**
1	38.74	38.74	38.74	38.70	38.72	38.70
2	30.91	30.90	30.91	.0.89	30.87	30.89

3	79.32	79.33	79.32	79.43	79.39	79.35
4	39.66	39.66	39.66	39.65	39.63	39.66
5	142.05	142.06	142.05	142.04	141.99	142.07
6	122.75	122.72	122.75	122.78	122.78	122.74
7	32.66	33.36	32.66	32.88	32.64	32.89
8	33.44	33.38	33.44	32.93	33.41	32.93
9	51.64	51.62	51.63	51.84	51.59	51.85
10	38.15	38.15	38.15	38.19	38.12	38.19
11	21.84	21.91	21.84	22.12	21.84	22.13
12	33.01	33.38	33.00	41.08	32.98	41.08
13	46.00	46.49	46.00	41.57	45.97	41.56
14	54.08	53.36	54.07	57.95	54.05	57.95
15	32.25	32.94	32.25	33.32	32.24	33.35
16	90.69	89.78	90.69	82.35	90.68	82.36
17	91.45	90.06	91.45	63.89	91.43	63.88
18	17.67	18.01	17.67	16.69	17.69	16.92
19	19.99	19.98	19.99	19.99	19.99	19.99
20	45.68	46.75	45.68	43.06	45.66	43.05
21	9.25	10.06	9.25	15.01	9.27	15.03
22	111.10	112.31	111.10	110.73	111.09	110.73
23	33.37	22.49	33.37	32.57	33.36	32.57
24	29.59	25.33	29.59	30.03	29.57	30.03
25	31.44	28.58	31.44	31.58	31.42	31.59
26	67.86	69.56	67.86	68.00	67.84	68.00
27	17.64	16.67	17.64	17.64	17.65	17.64

Table S2 ^{13}C-NMR data of the sugar portion of compounds 5-10

Sugar portion		Compounds					
		**5**	**6**	**7**	**8**	**9**	**10**
Glc	1	100.66	100.67	100.66	100.60	100.56	100.68

	2	79.18	79.18	79.54	79.45	79.46	79.18
	3	77.89	79.53	79.18	80.13	76.70	77.86
	4	79.54	77.88	77.89	76.72	80.06	79.52
	5	71.99	72.00	71.99	78.18	78.15	71.98
	6	62.91	62.91	62.91	62.09	62.07	62.91
Rha I	1	102.33	102.31	102.33	102.43	102.43	102.31
	2	72.38	72.37	72.38	72.33	72.30	72.36
	3	72.53	72.54	72.53	79.42	72.49	72.53
	4	74.09	74.10	74.09	73.87	74.04	74.07
	5	69.90	69.90	69.90	69.91	69.91	69.89
	6	18.11	18.11	18.11	18.13	18.02	18.11
Rha II	1				103.15	103.13	
	2				74.07	72.57	
	3				72.59	72.30	
	4				72.51	73.85	
	5				70.82	70.79	
	6				18.01	18.12	

Reference

1. Xie, B.B; Liu, H.Y; Ni, W; Chen, C.X. Ypsilandrosides C-G, five new spirostanol saponins from *Ypsilandra thibetica*. *Steroids* **2009**, *12*, 950-955.
2. Kang, L.P; Huang, Y.Y; Zhan, Z.L; Liu, D.H; Peng, H.S; Nan, T.G; Zhang, Y; Hao, Q.X; Tang, J.F; Zhu, S.D; et al. Structural characterization and discrimination of the *Paris polyphylla* var. *yunnanensis* and *Paris vietnamensis* based on metabolite profiling analysis. *Journal of Pharmaceutical and Biomedical Analysis* **2017**, *142*, 252-261.
3. Tang, L.Y; Wang, Z.J; Wu, H.W; Yokosuka, A; Mimaki, Y. Steroidal glycosides from the underground parts of *Dracaena thalioides* and their cytotoxic activity. *Phytochemistry* **2014**, *107*, 102-110.
4. Khodakov, G.V; Akimov, Y.A; Shashkov, A.S; Kintia, P.K; Grishkovets, V.I. Triterpene and steroid saponins isolated from two *Melilotus* species. *Oxygen*

Transport to Tissue XXXIII **1996**, *405*,: 211-222.

5. Li, Y.H; Liu, C.X; Xiao, D; Han, J; Yue, Z.G; Sun, Y; Fan, L; Zhang, F; Meng, J; Zhang, R; et al. *Trillium tschonoskii* steroidal saponins suppress the growth of colorectal Cancer cells in vitro and in vivo. *Journal of Ethnopharmacology* **2015**, *168*, 136-145.
6. Lee, H.J; Watanabe, B; Nakayasu, M; Onjo, M; Sugimoto, Y; Mizutani, M. Novel steroidal saponins from *Dioscorea esculenta* (Togedokoro). *Bioscience, Biotechnology, and Biochemistry* **2017**, *81*, 2253-2260.

S1. Positive HR-ESI-MS spectrum of compound **1**

Mass	Calc. Mass	mDa	PPM	DBE	i-FIT	Formula
803.4170	803.4194	-2.4	-3.0	9.5	212.5	$C_{41}H_{64}O_{14}Na$

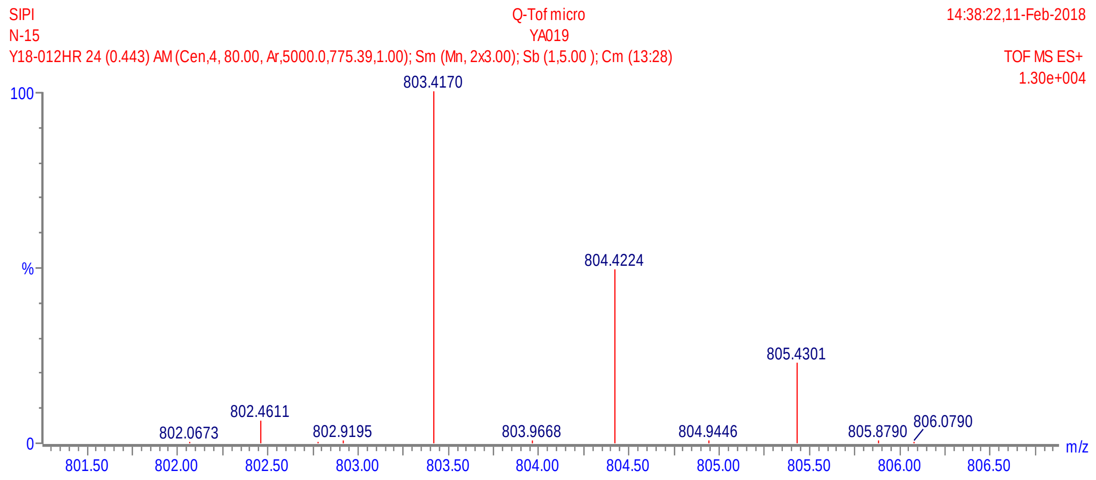

S2. Positive and Negative ESI-MS spectrum of compound **1**

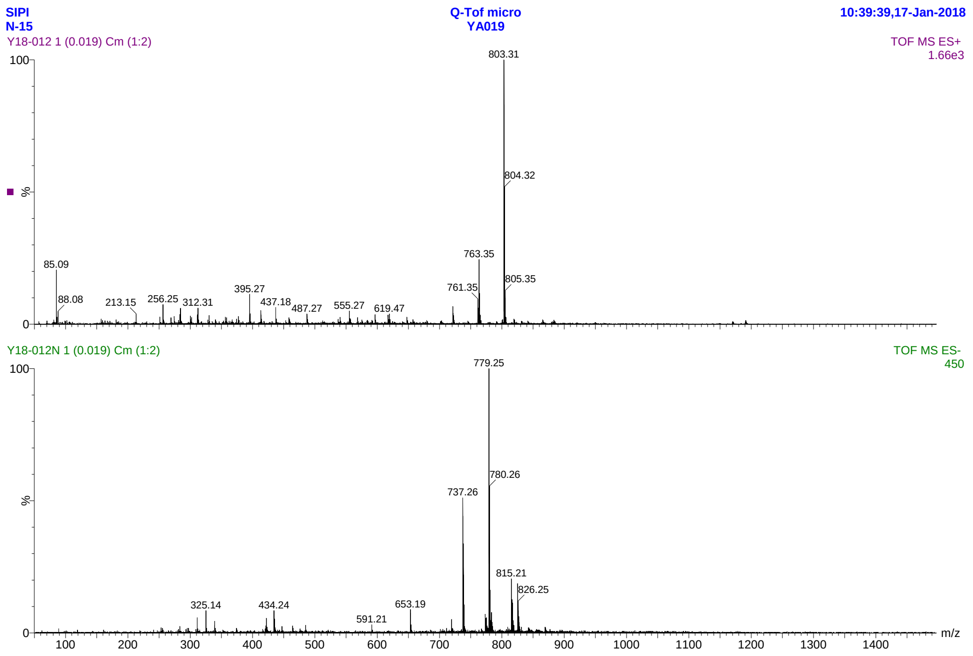

S3. ^{1}H NMR (800 MHz, CD_3OD) spectrum of compound **1**

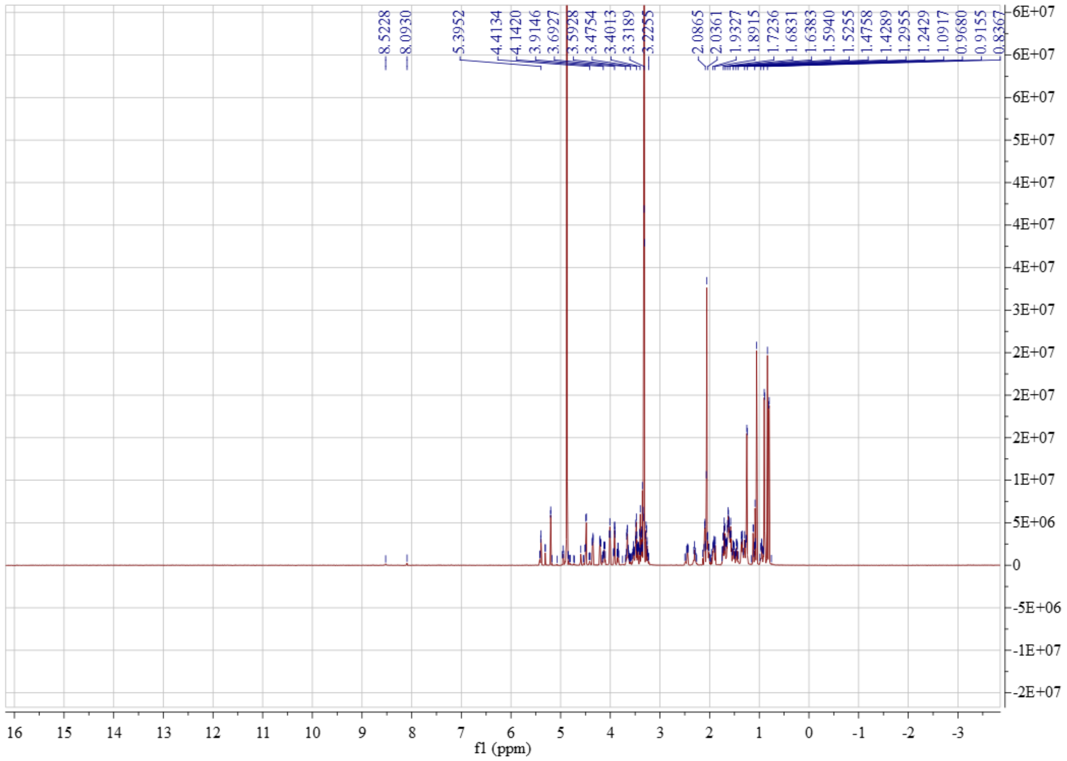

S4. ^{13}C NMR (201 MHz, CD_3OD) spectrum of compound **1**

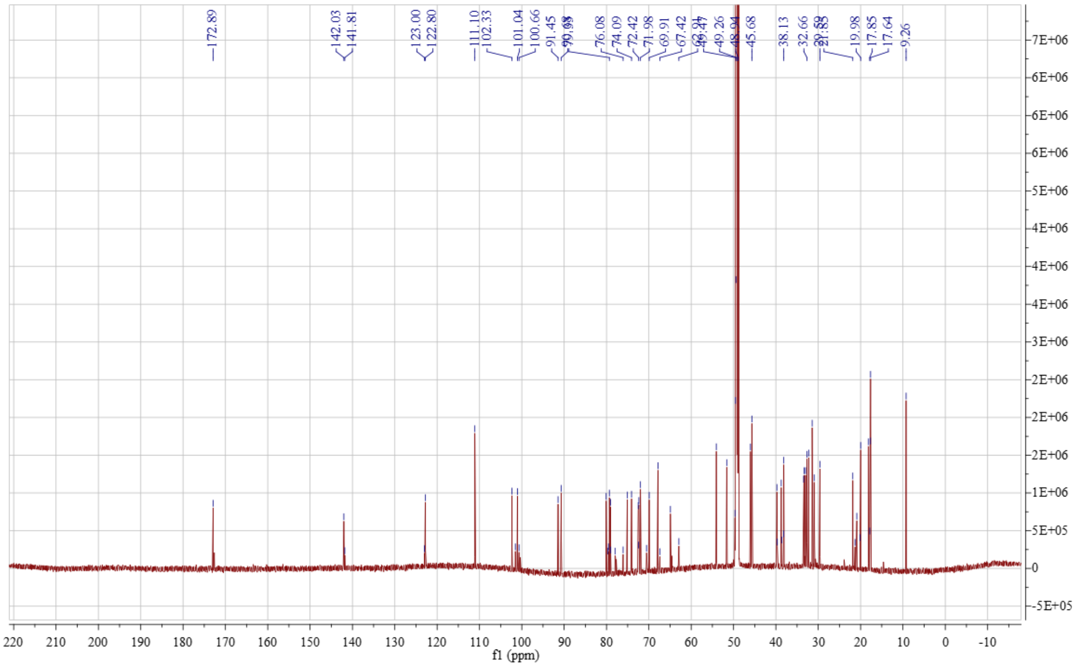

S5. DEPT 135 spectrum of compound **1**

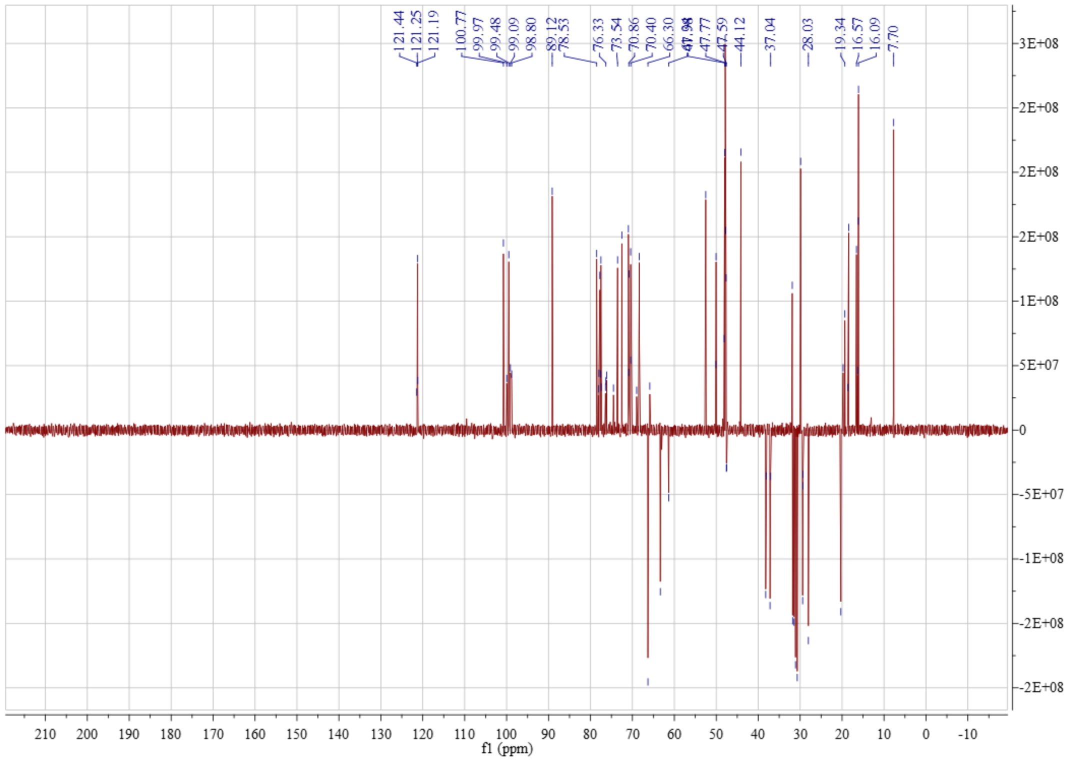

S6. HSQC spectrum of compound **1**

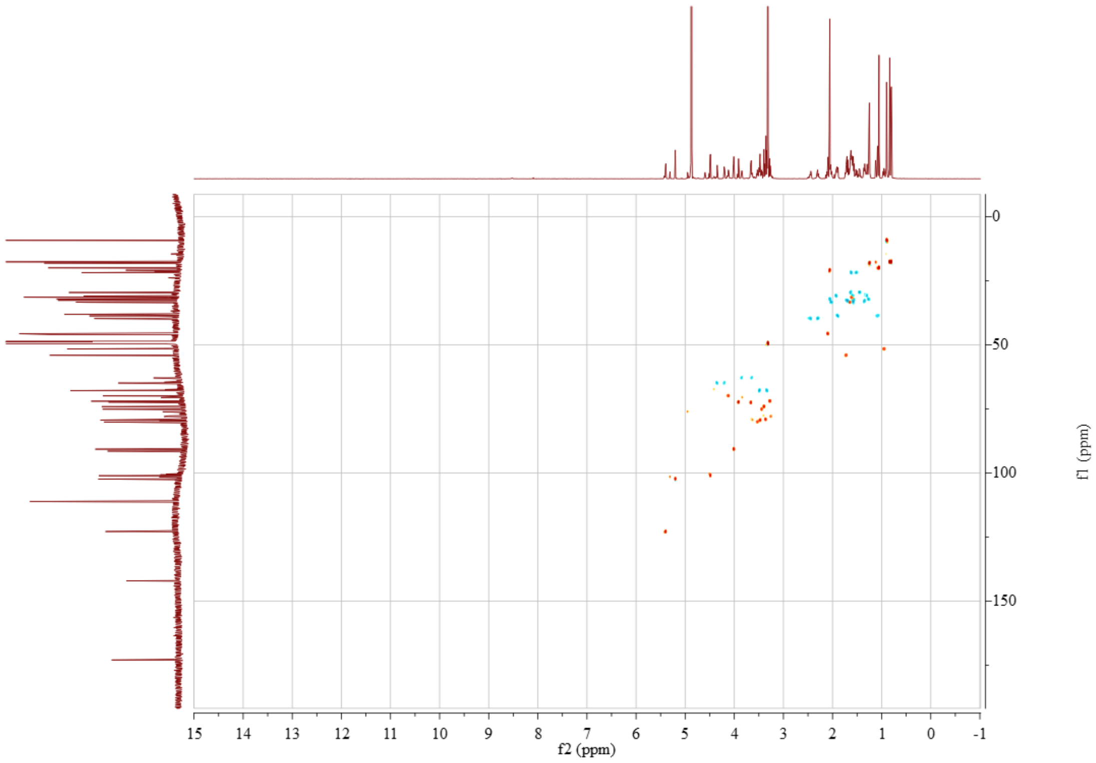

S7. HMBC spectrum of compound **1**

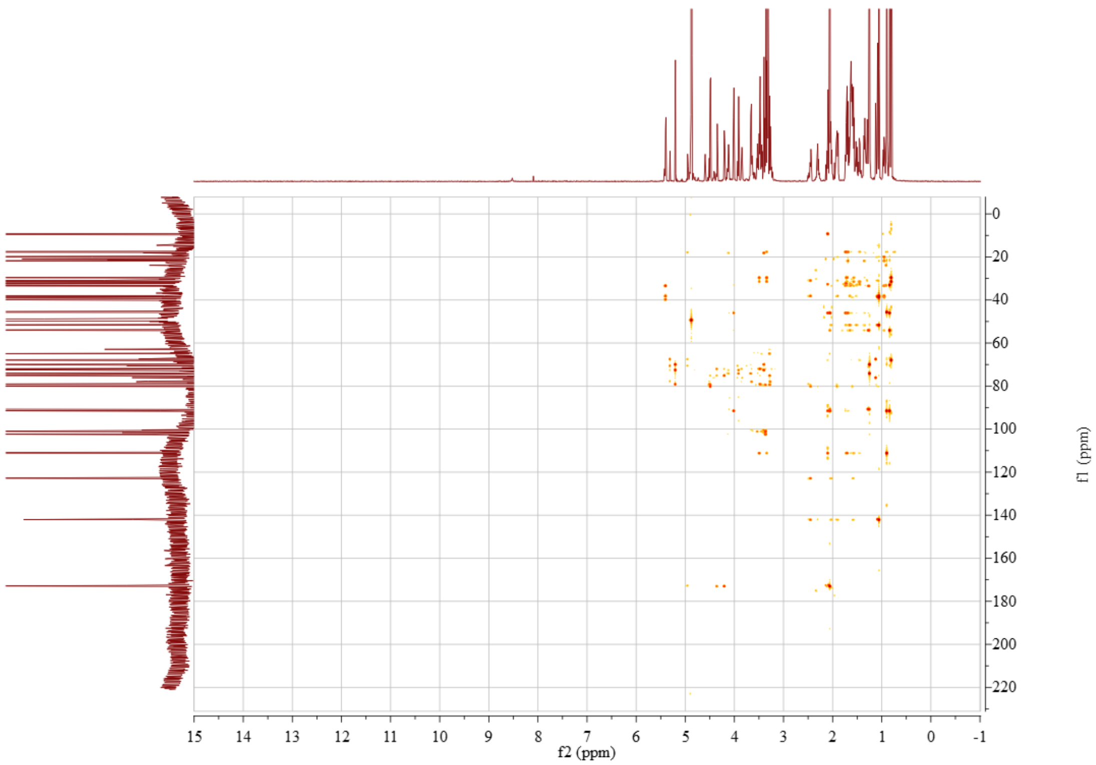

S8. ^{1}H-^{1}H COSY spectrum of compound **1**

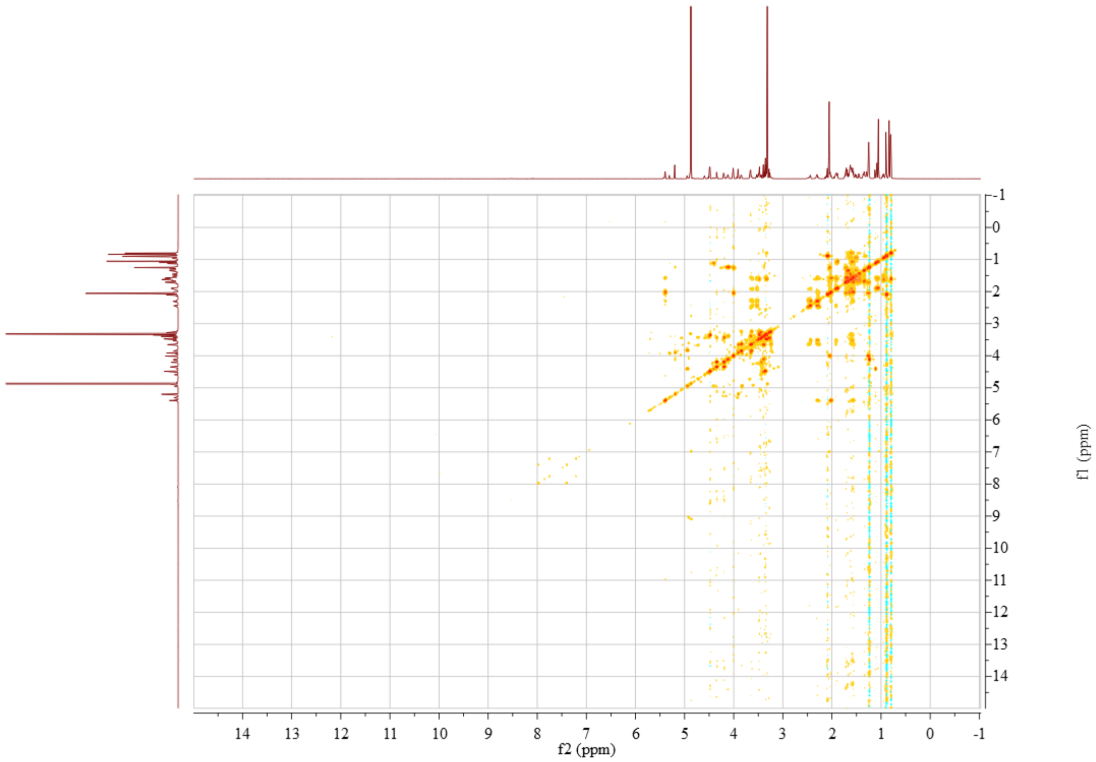

S9. NOESY spectrum of compound **1**

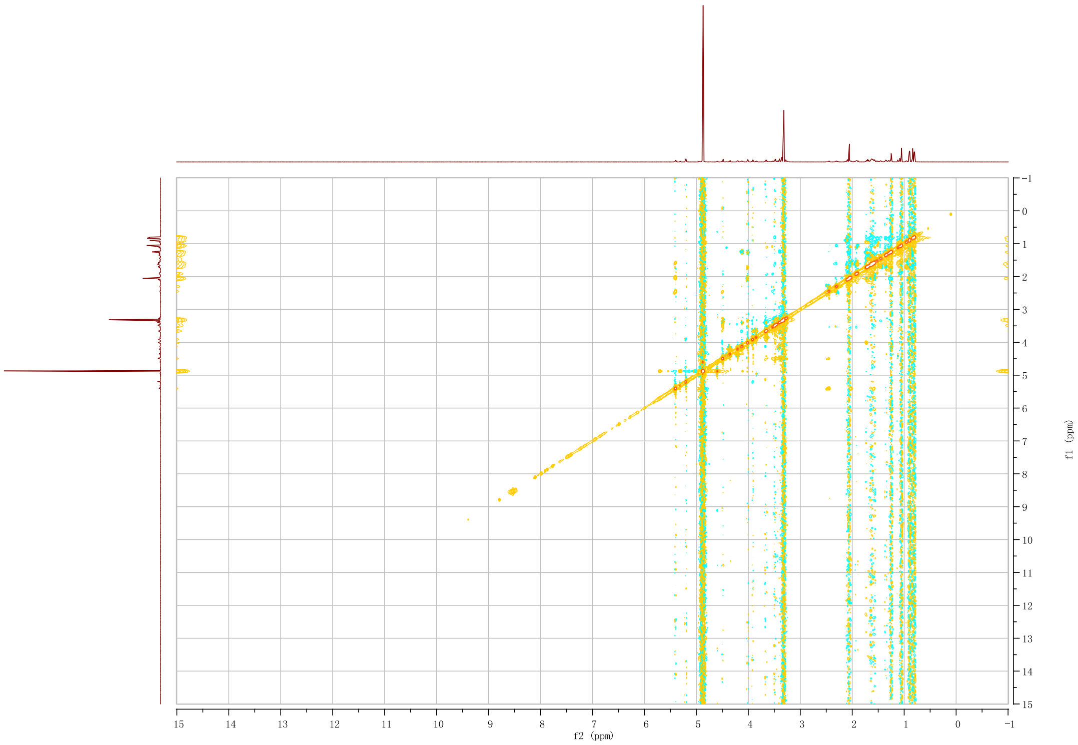

S10. TOCSY spectrum of compound **1**

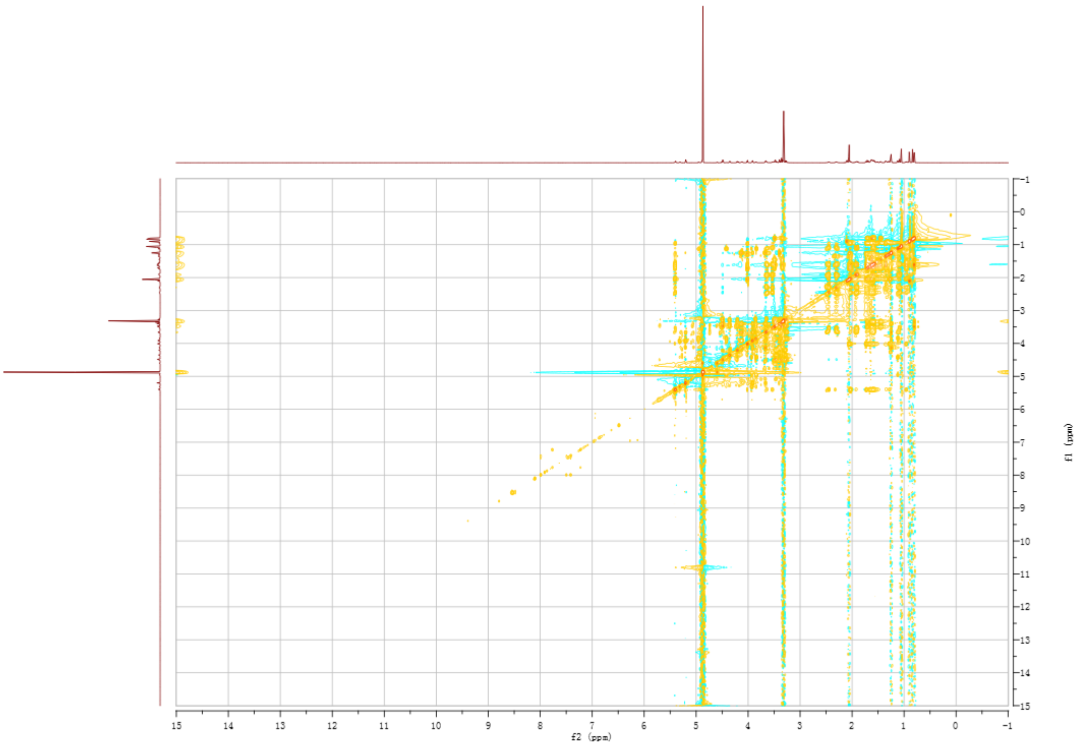

S11. Positive HR-ESI-MS spectrum of compound **2**

Mass	Calc. Mass	mDa	PPM	DBE	i-FIT	Formula
777.4044	777.4037	0.7	0.9	8.5	140.1	$C_{39}H_{62}O_{14}Na$

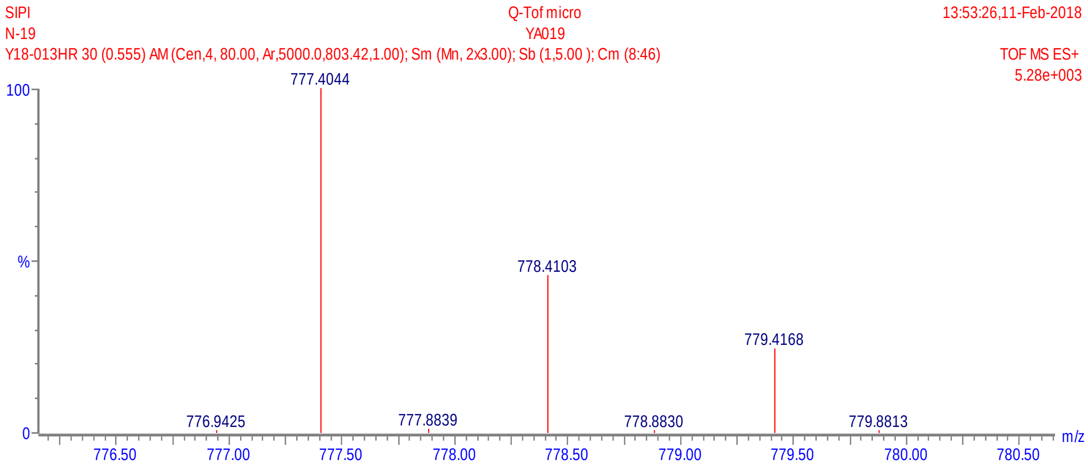

S12. Positive and Negative ESI-MS spectrum of compound **2**

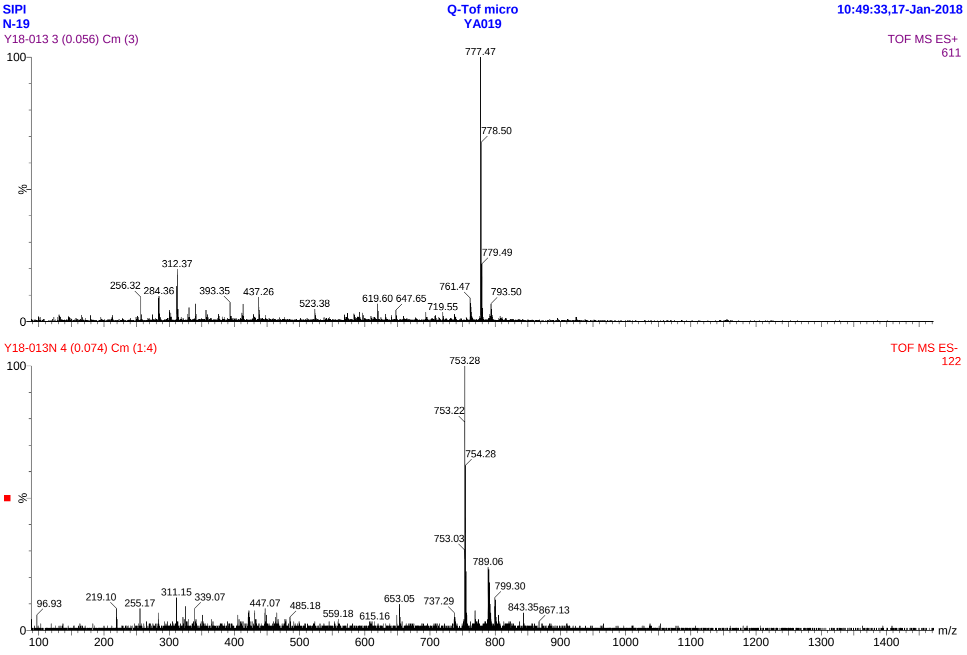

S13. ^{1}H NMR (800 MHz, CD_3OD) spectrum of compound **2**

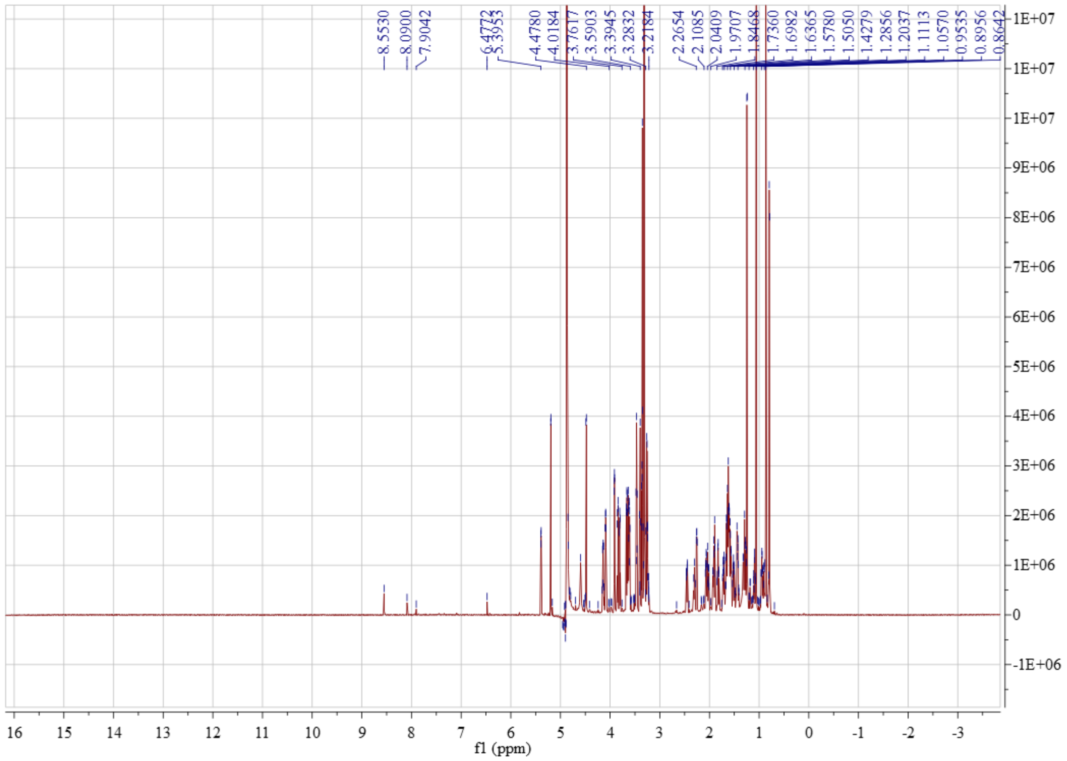

S14. ^{13}C NMR (201 MHz, CD_3OD) spectrum of compound **2**

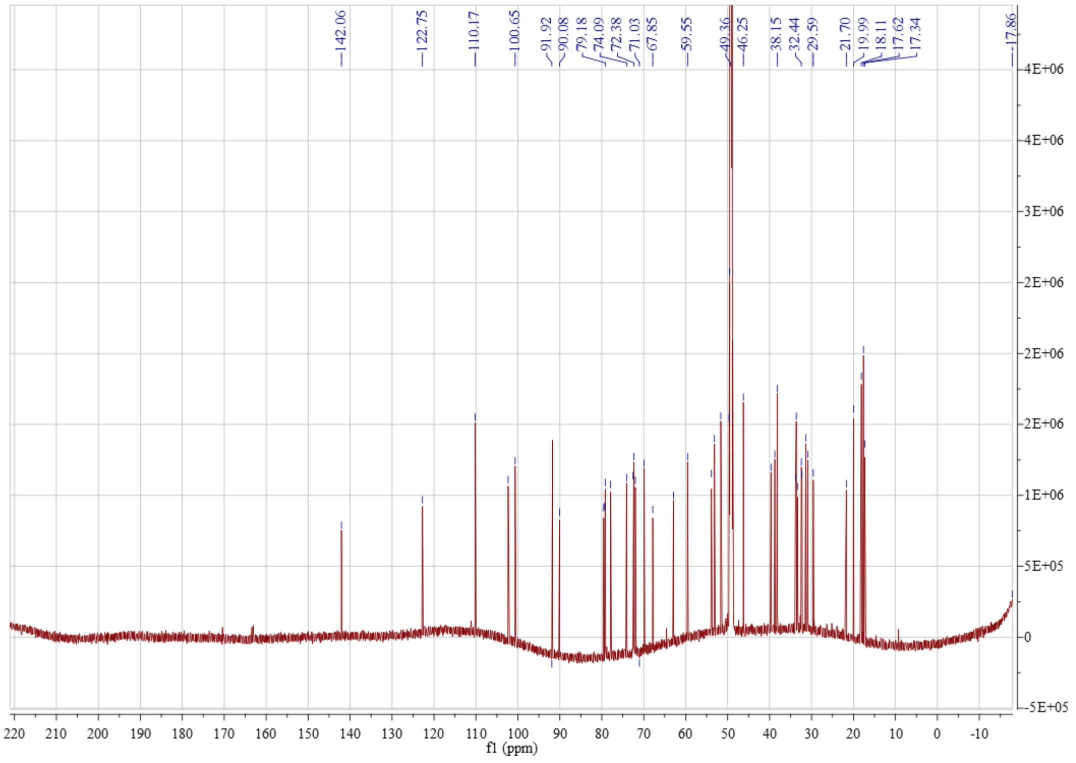

S15. DEPT 135 spectrum of compound **2**

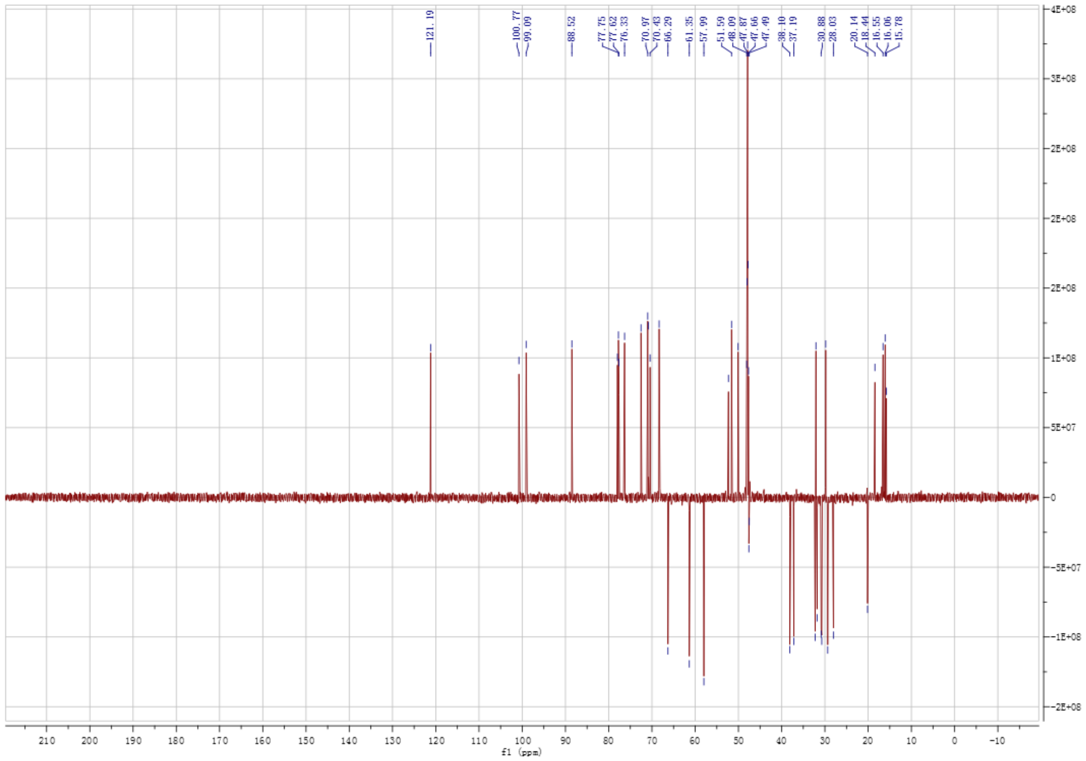

S16. HSQC spectrum of compound **2**

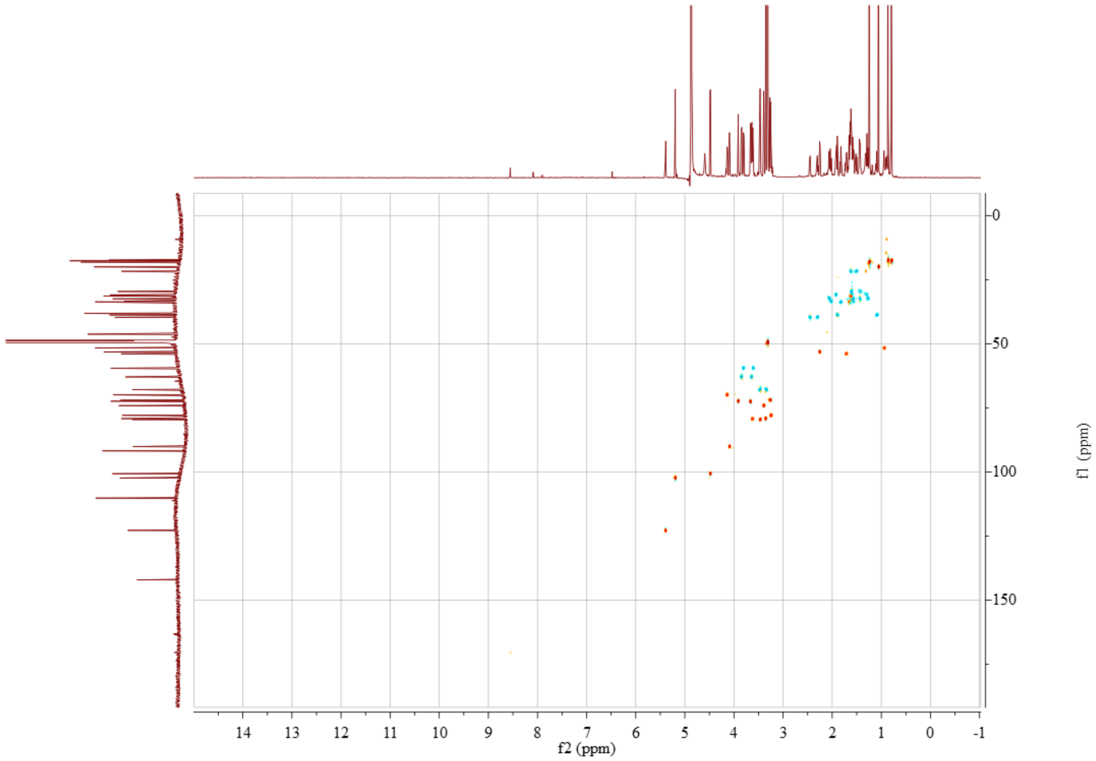

S17. HMBC spectrum of compound **2**

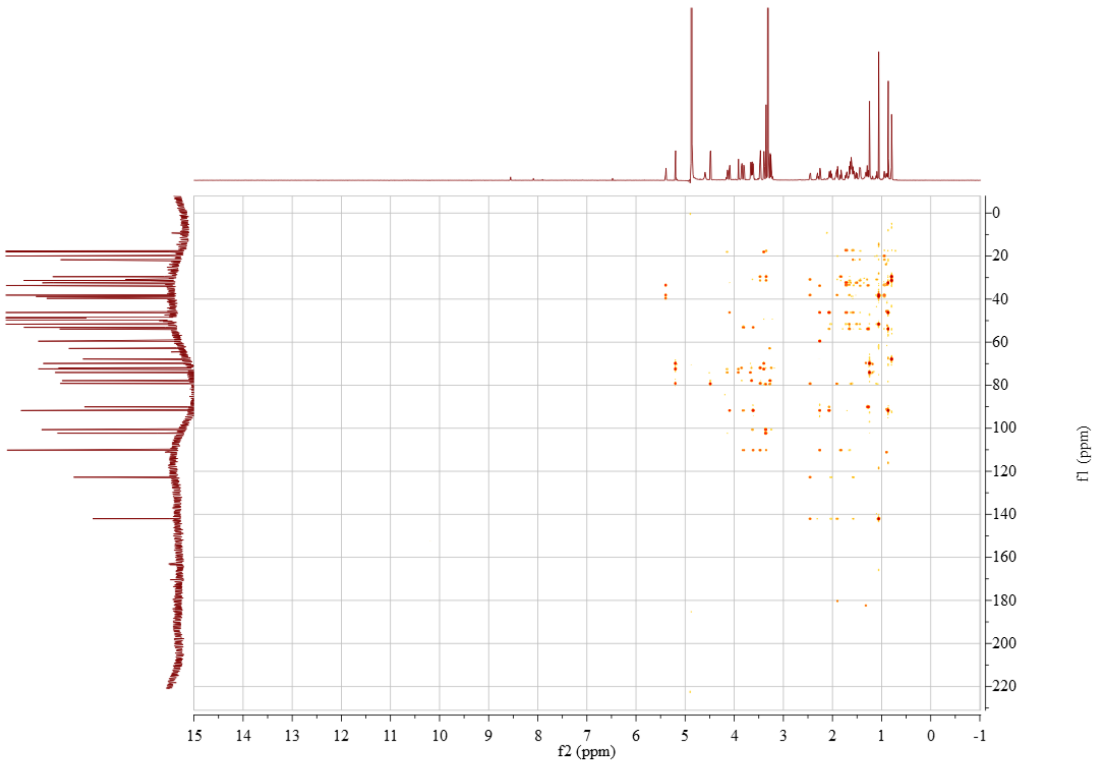

S18. ^{1}H-^{1}H COSY spectrum of compound **2**

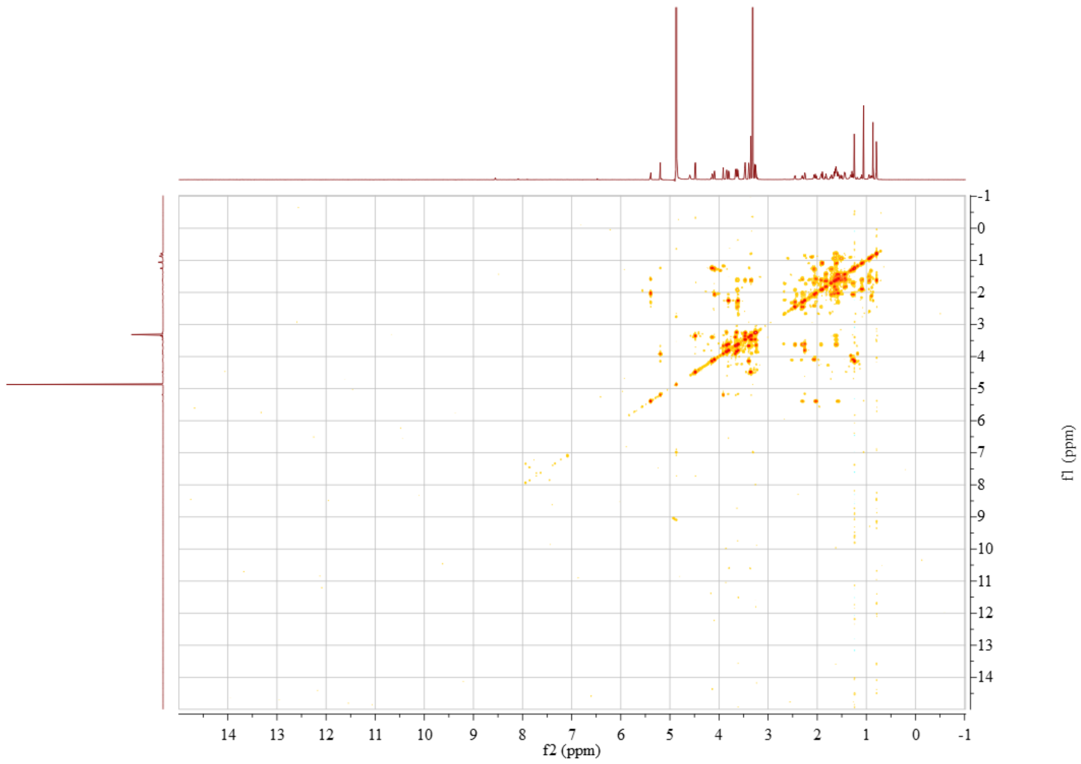

S19. NOESY spectrum of compound **2**

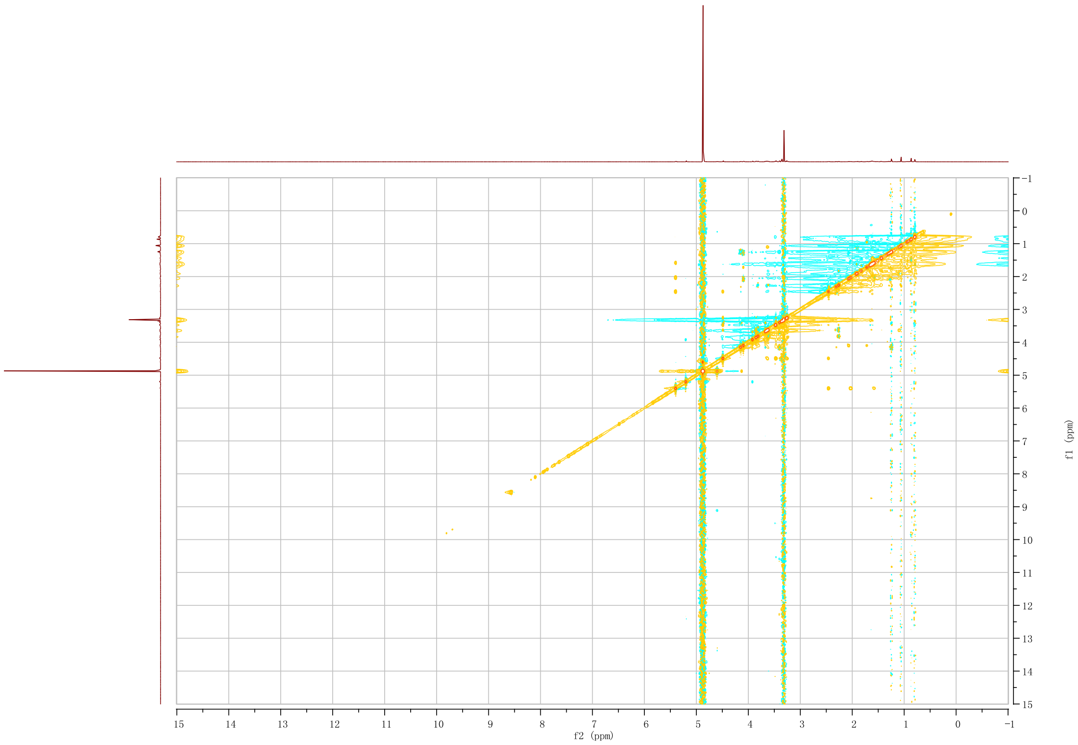

S20. TOCSY spectrum of compound **2**

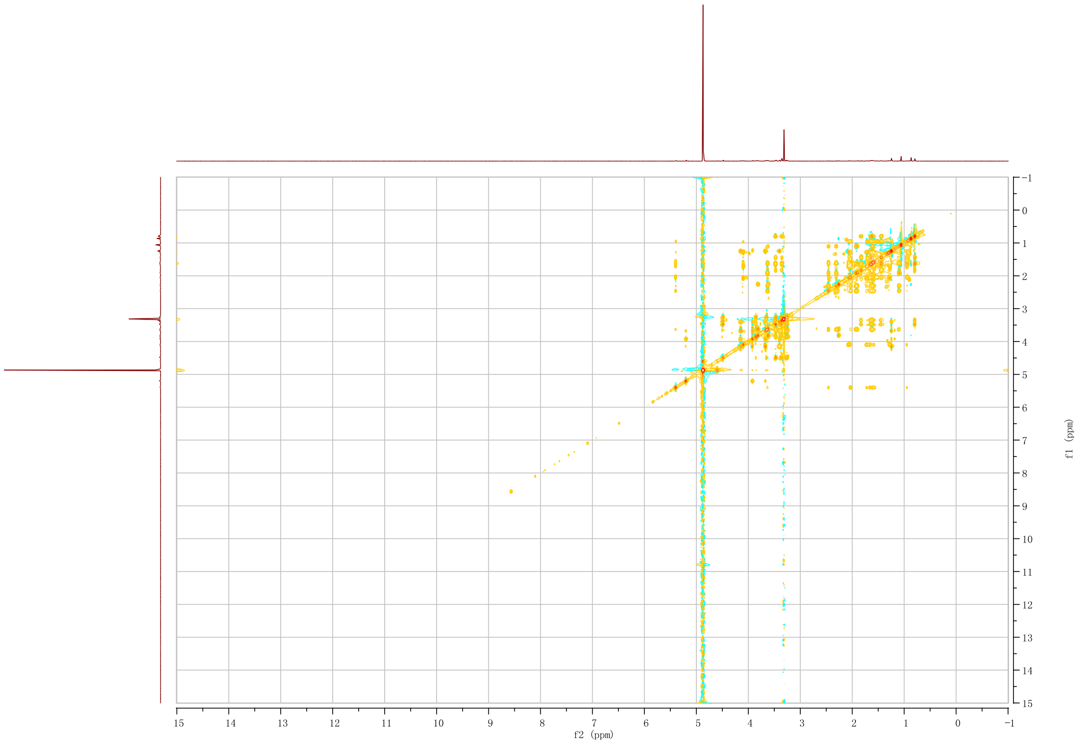

S21. Positive HR-ESI-MS spectrum of compound **3**

Mass	Calc. Mass	mDa	PPM	DBE	i-FIT	Formula
775.3884	775.3881	0.3	0.4	9.5	n/a	$C_{39}H_{60}O_{14}Na$

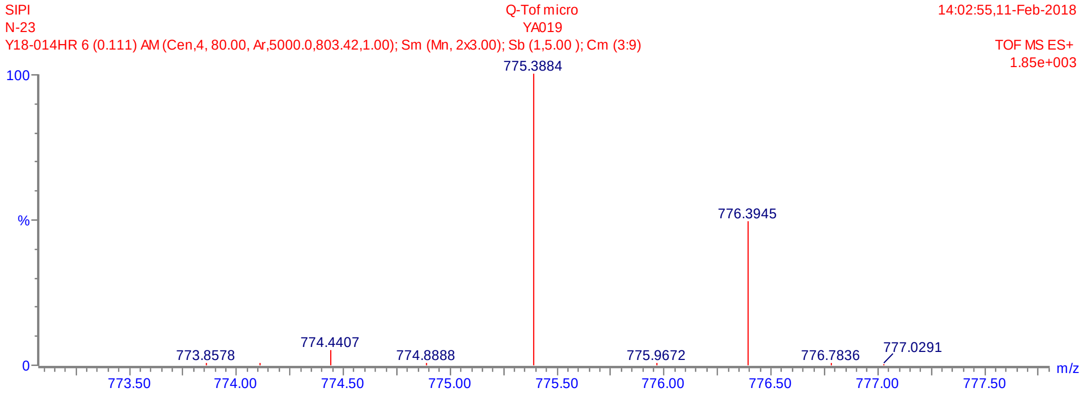

S22. Positive ESI-MS spectrum of compound **3**

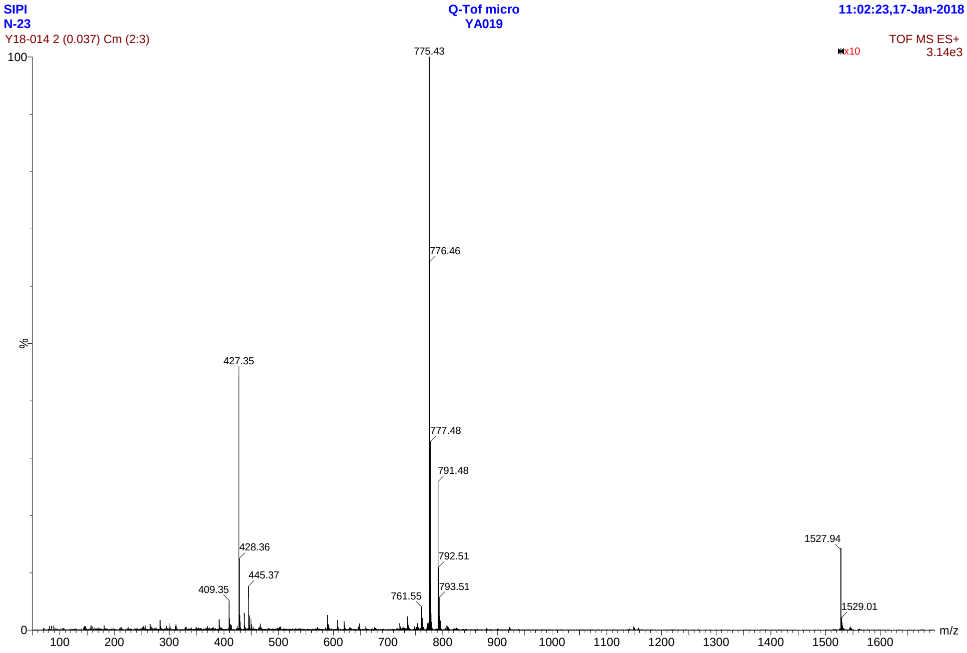

S23. ^{1}H NMR (500 MHz, CD_3OD) spectrum of compound **3**

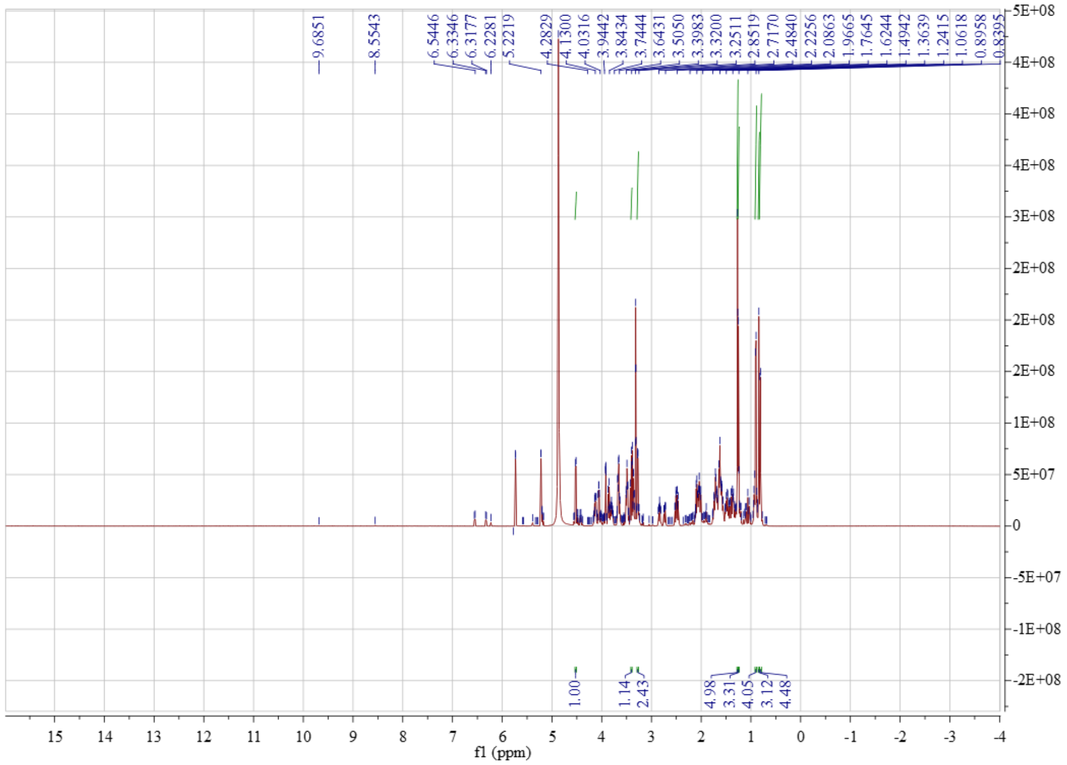

S24. ^{13}C NMR (125 MHz, CD_3OD) spectrum of compound **3**

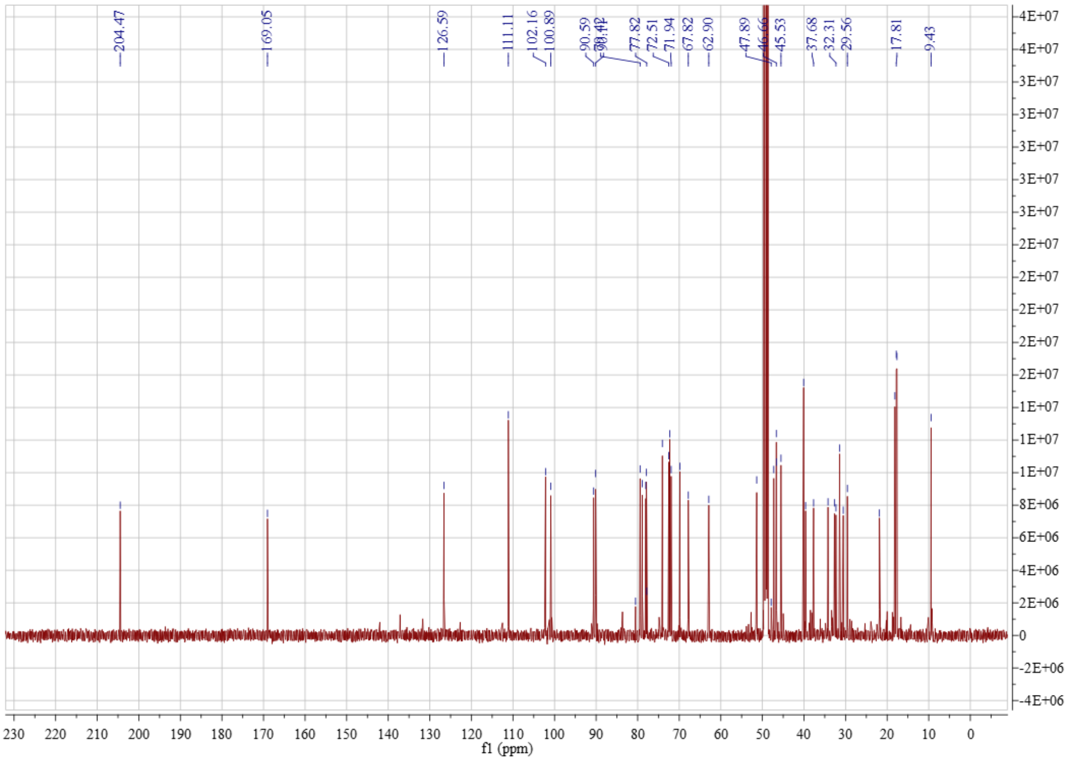

S25. DEPT 135 spectrum of compound **3**

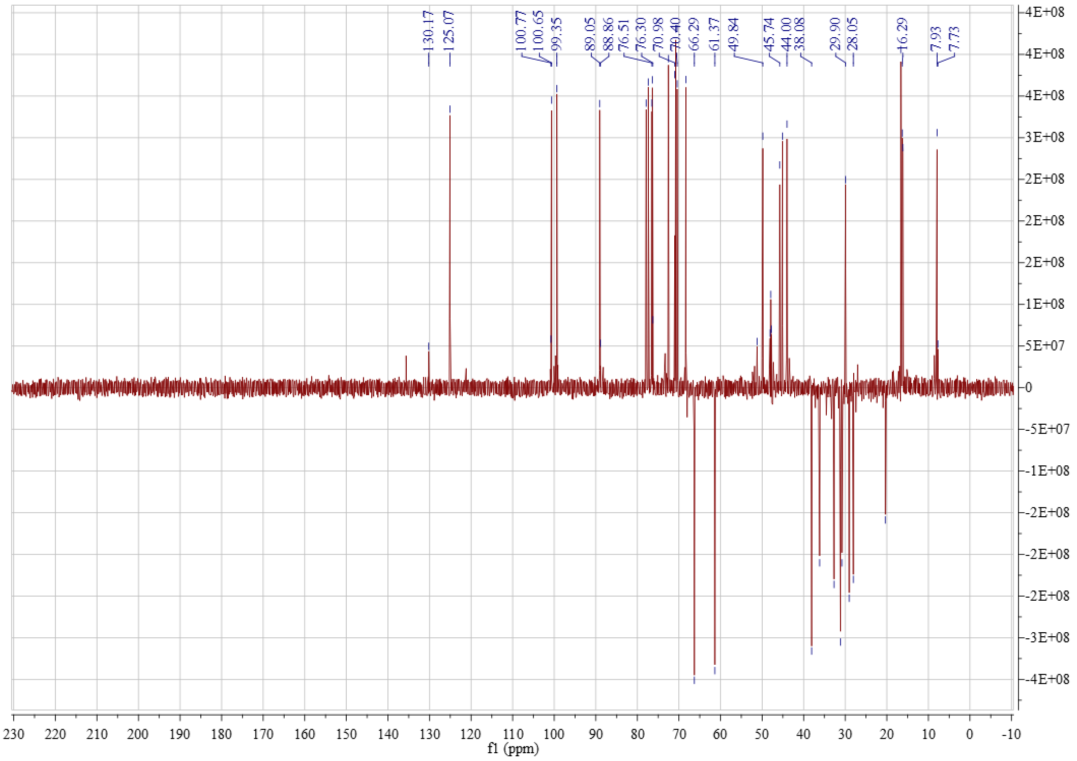

S26. HSQC spectrum of compound **3**

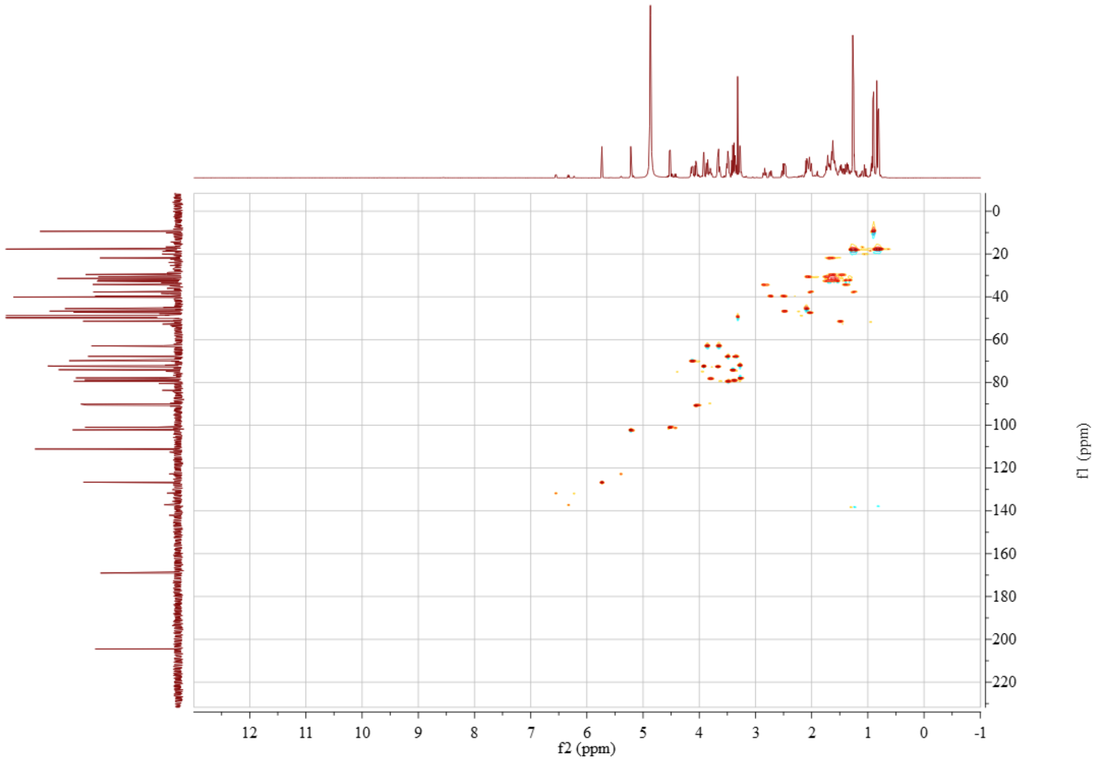

S27. HMBC spectrum of compound **3**

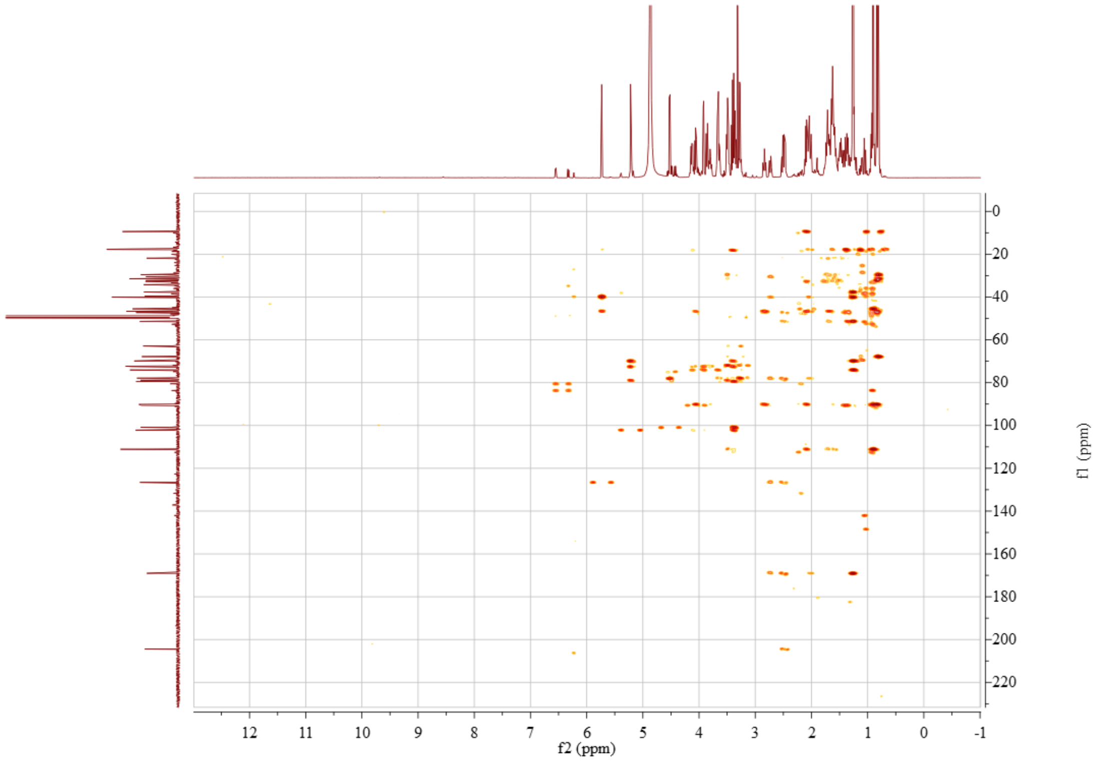

S28. ^{1}H-^{1}H COSY spectrum of compound **3**

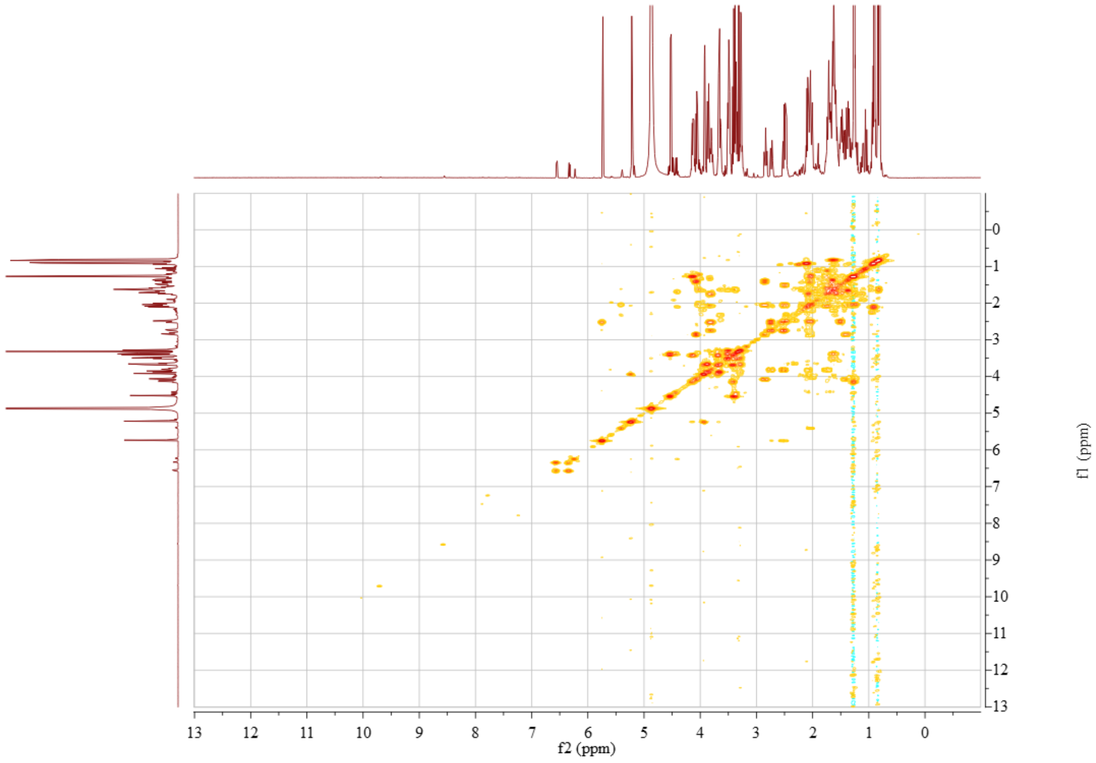

S29. NOESY spectrum of compound **3**

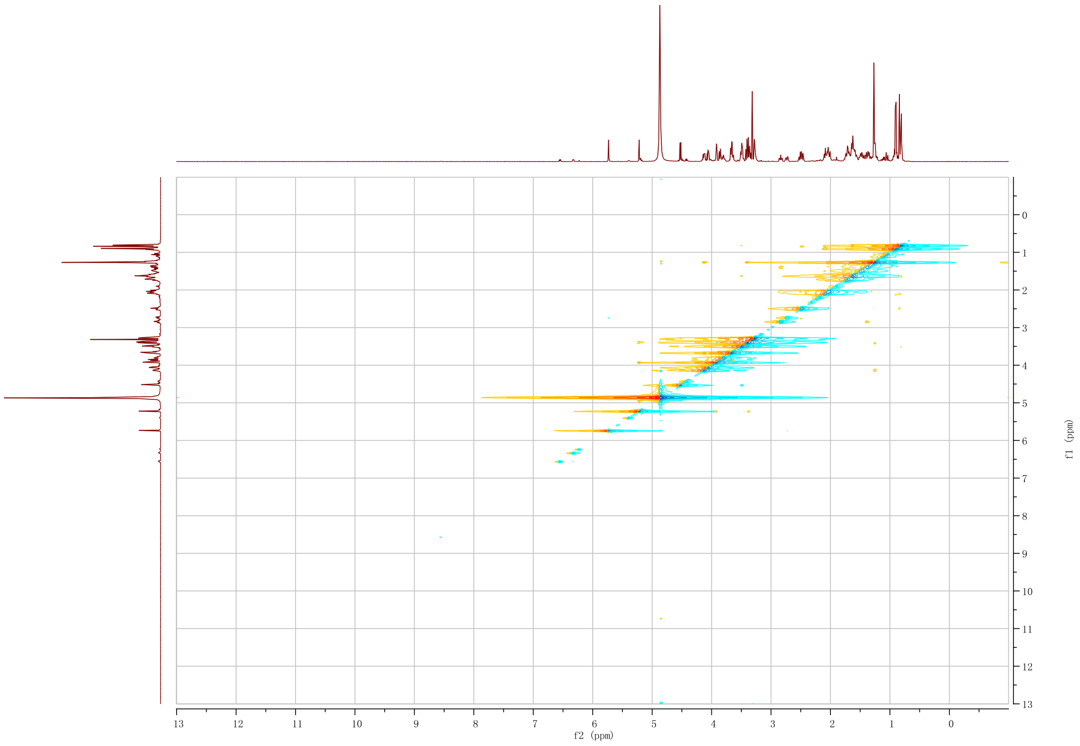

S30. TOCSY spectrum of compound **3**

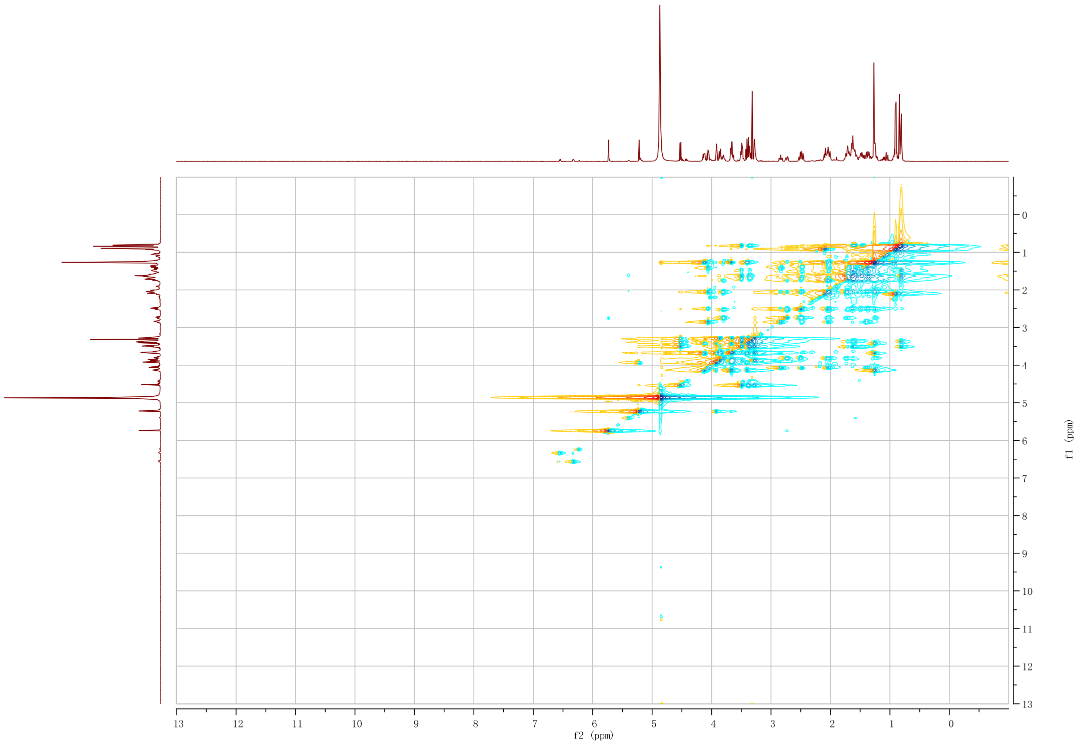

S31. Positive HR-ESI-MS spectrum of compound **4**

Mass	Calc. Mass	mDa	PPM	DBE	i-FIT	Formula
775.3849	775.3881	-3.2	-4.1	9.5	36.7	$C_{39}H_{60}O_{14}Na$

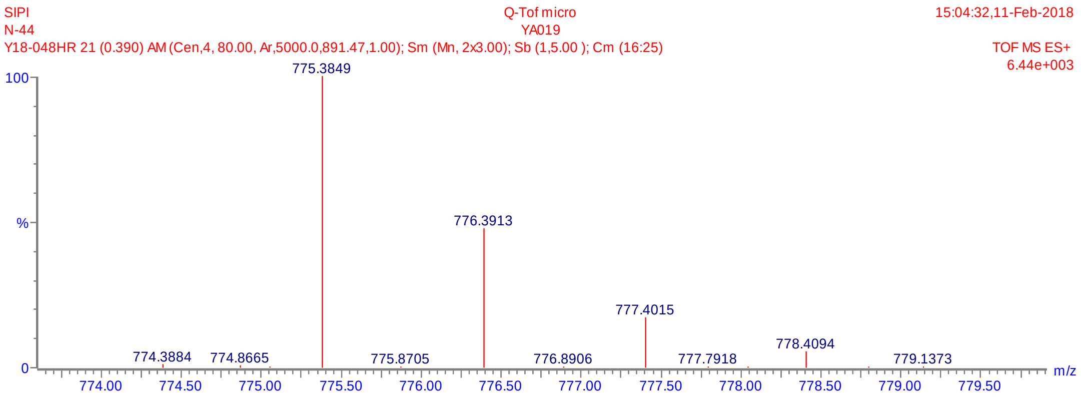

S32. ^{1}H NMR (500 MHz, CD_3OD) spectrum of compound **4**

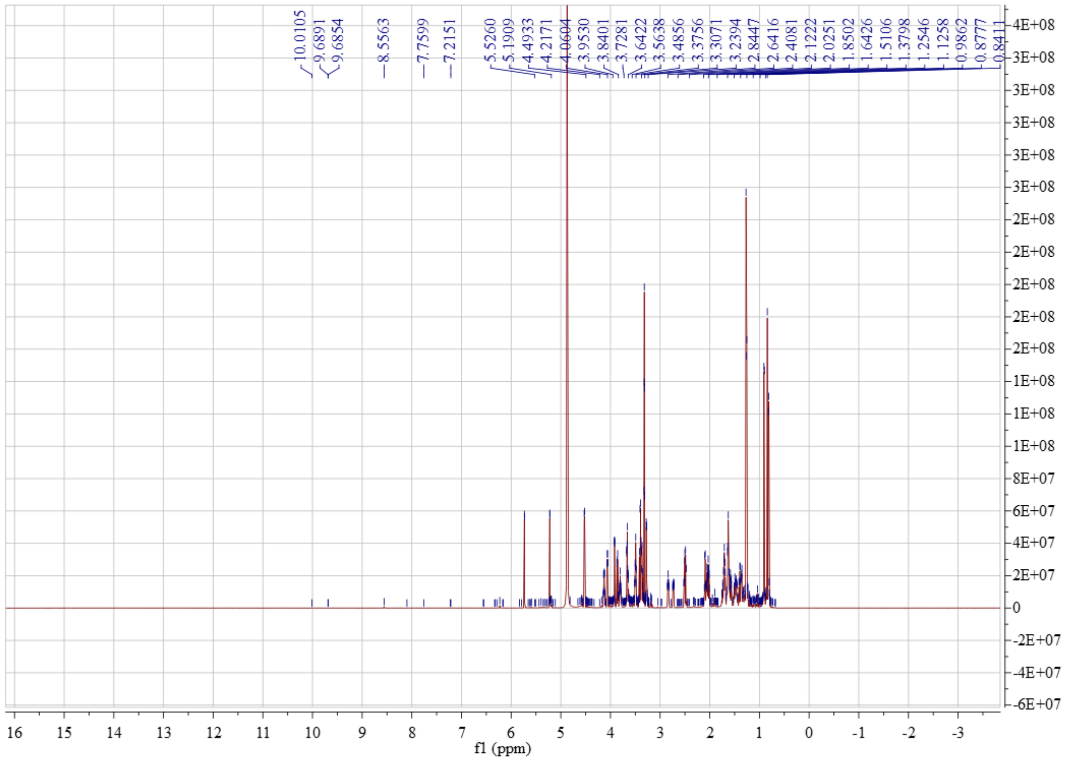

S33. ^{13}C NMR (125 MHz, CD_3OD) spectrum of compound **4**

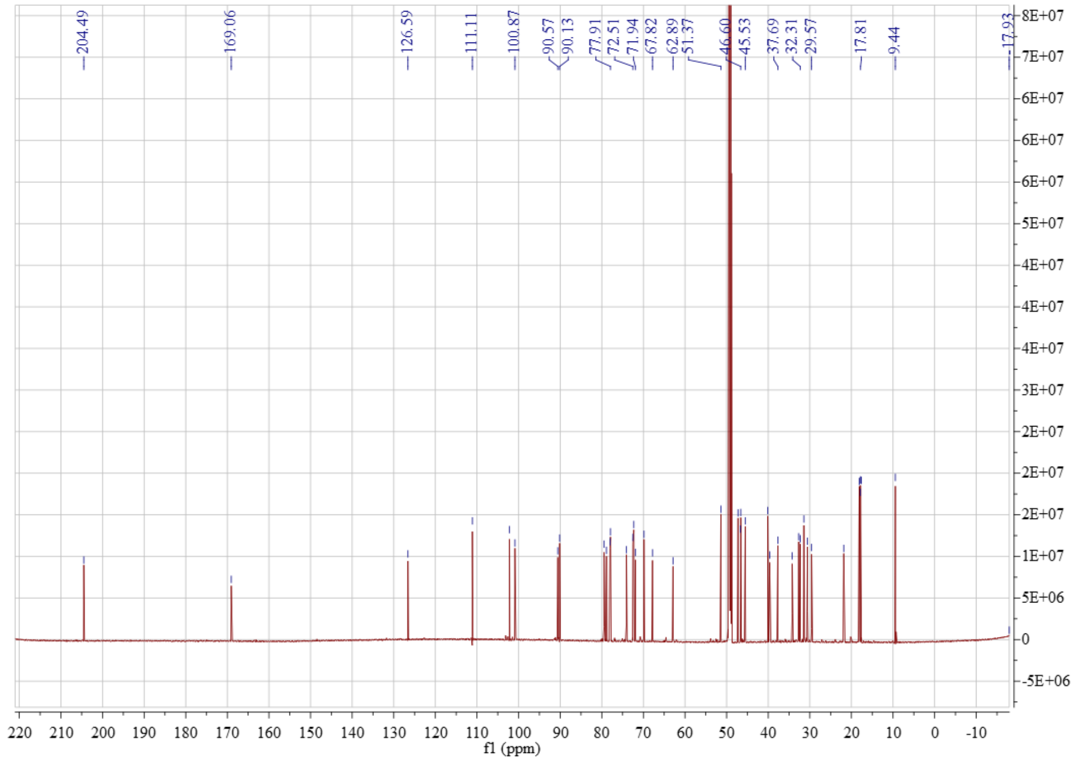

S34. DEPT 135 spectrum of compound **4**

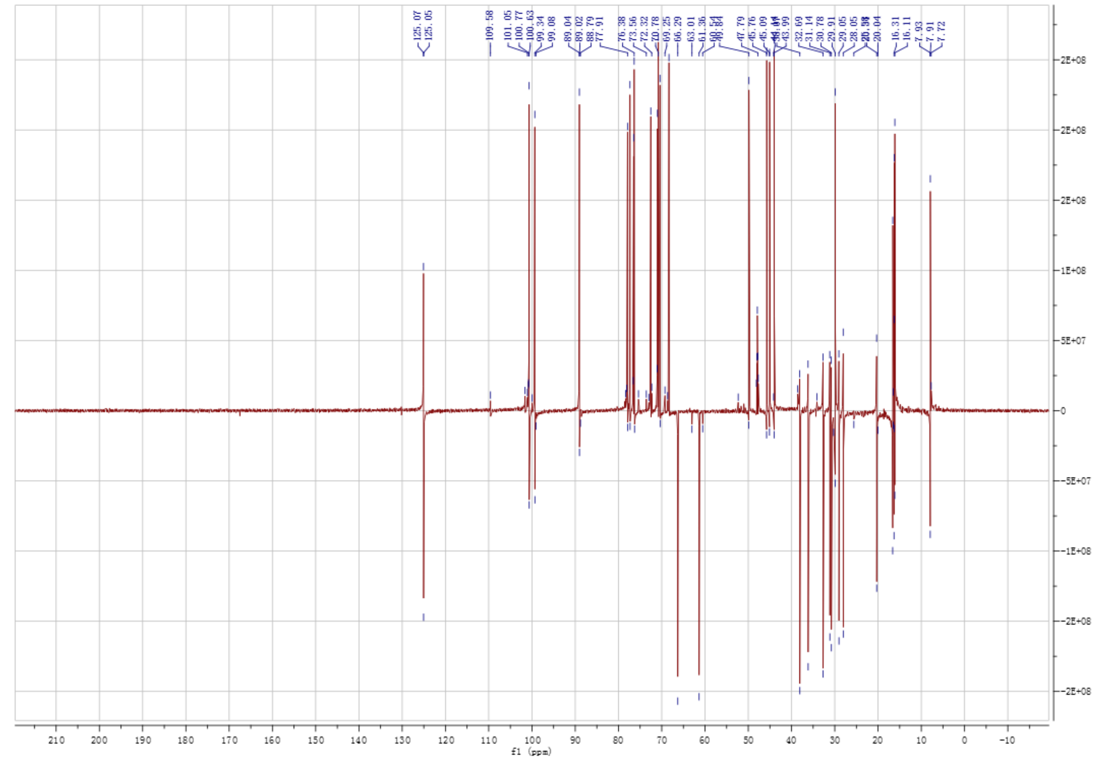

S35. HSQC spectrum of compound **4**

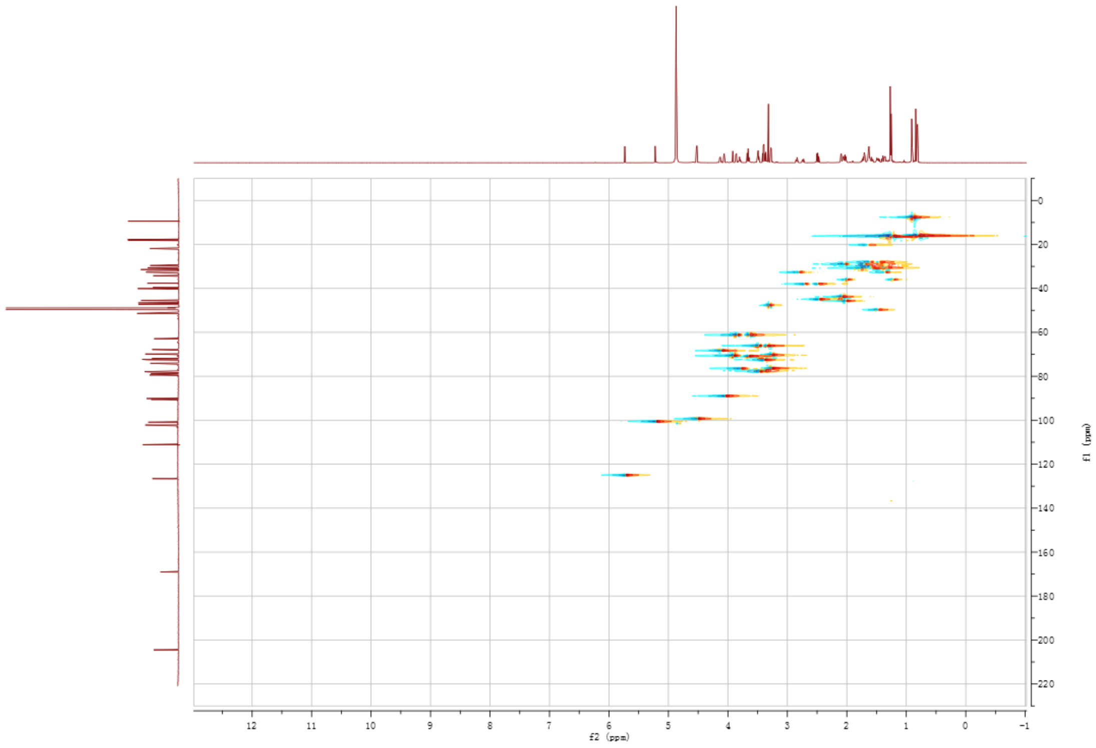

S36. HMBC spectrum of compound **4**

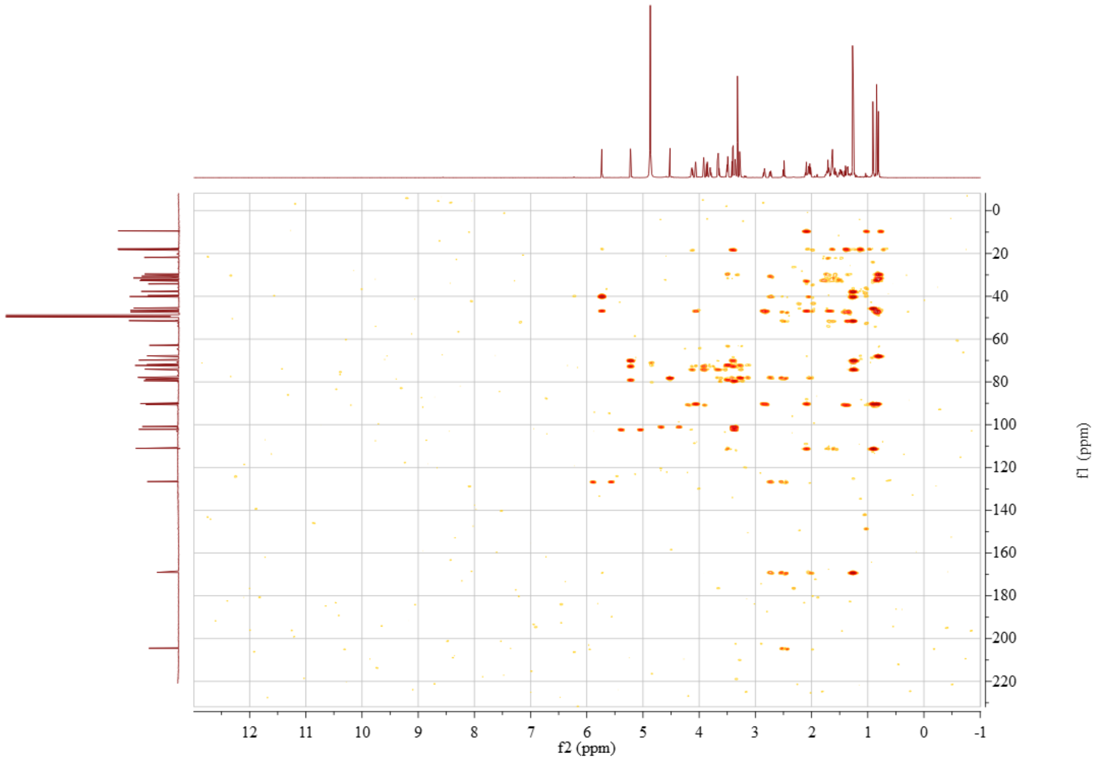

S37. ^{1}H-^{1}H COSY spectrum of compound **4**

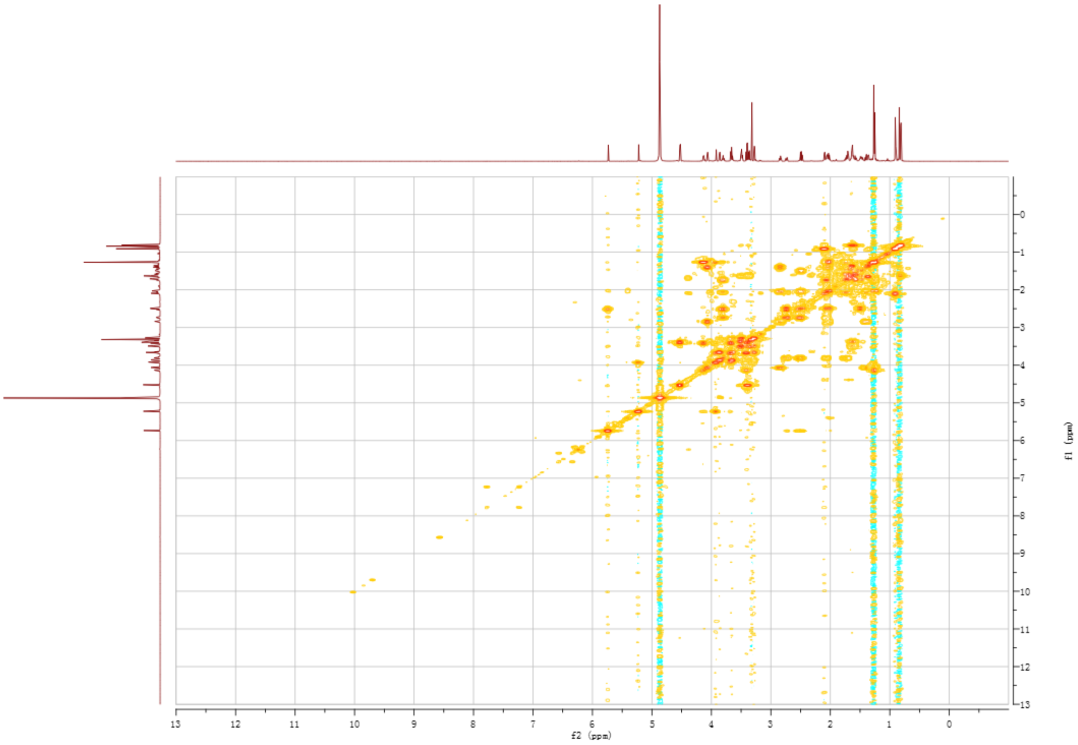

S38. NOESY spectrum of compound **4**

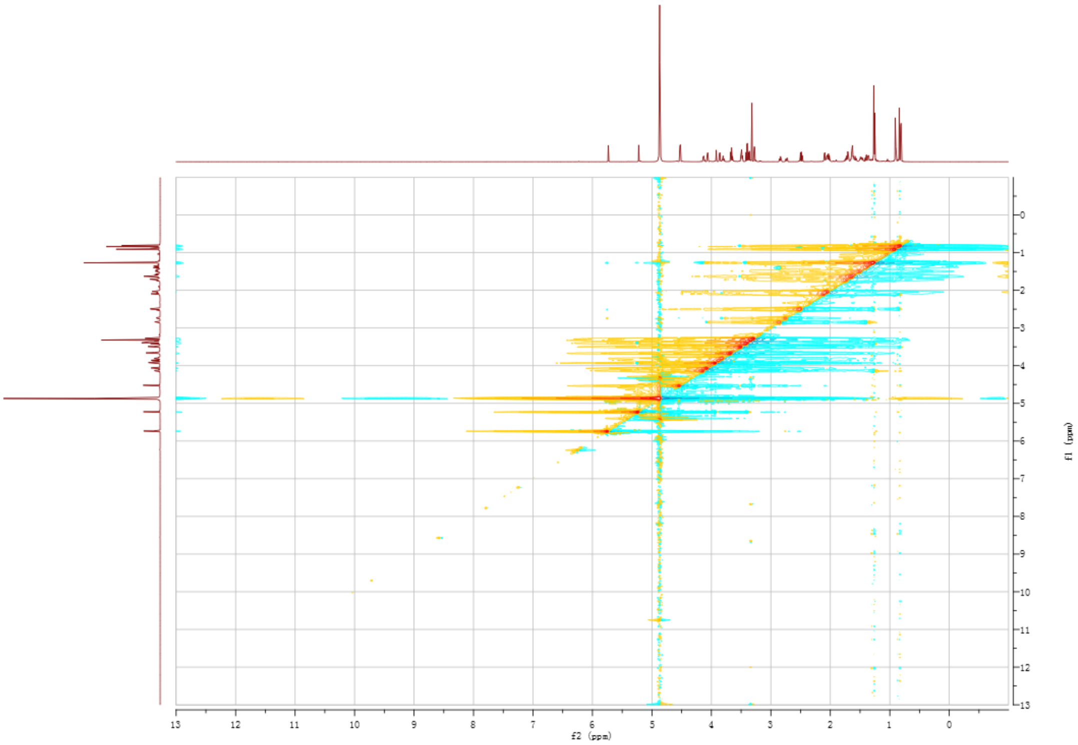

S39. TOCSY spectrum of compound **4**

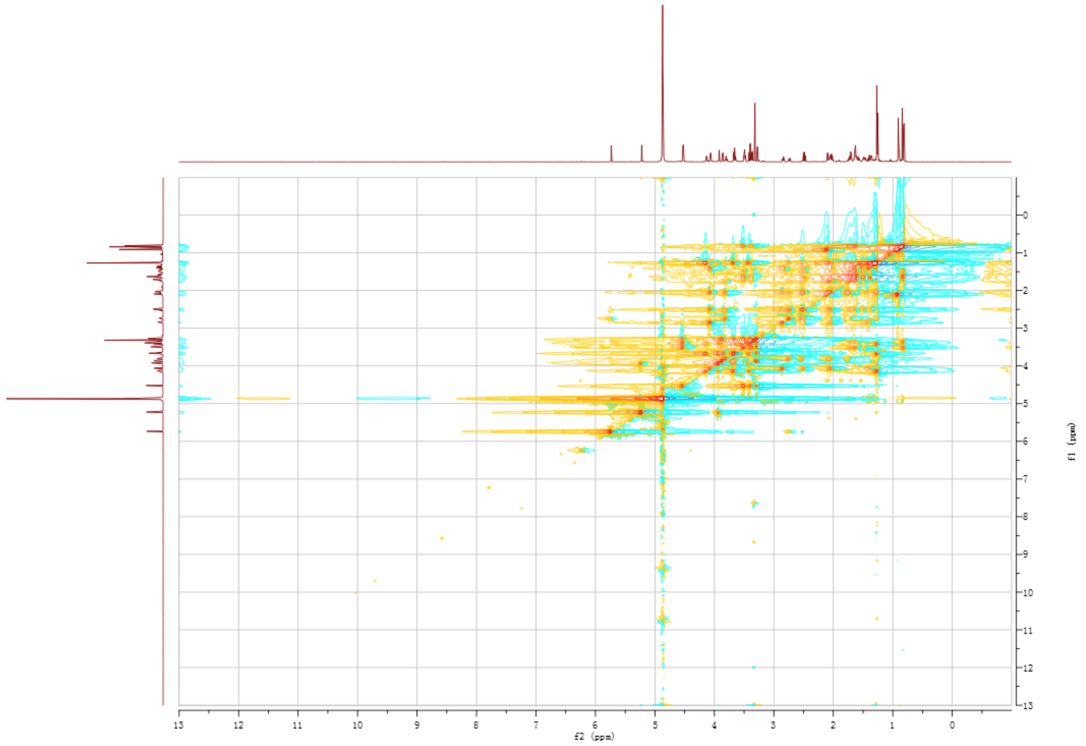

f2 (ppm)
f1 (ppm)
